# Flexoelectricity in Photoconversion: Fundamentals, Materials, and Outlooks

**DOI:** 10.1002/advs.75706

**Published:** 2026-05-20

**Authors:** Xiang Huang, Feng Li, Rongkun Zheng

**Affiliations:** ^1^ School of Physics The University of Sydney New South Wales Australia

## Abstract

Flexoelectricity, the generation of electric polarization by a strain gradient, has recently emerged as a powerful tool for manipulating photoconversion processes in a wide range of materials. This review systematically examines how strain‐gradient‐induced flexoelectric fields can enhance and tailor both photovoltaic and photoconductive responses, overcoming fundamental limitations of conventional optoelectronic designs. We review key advances across material platforms, including oxide and halide perovskites, two‐dimensional semiconductors, with emphasis on mechanisms such as band‐structure modulation, carrier separation, and photoconductance tuning under mechanical strain. Experimental demonstrations of giant flexo‐photovoltaic coefficients, strain‐programmable photodetectors, and flexo‐enhanced photocatalytic systems are then surveyed, alongside theoretical insights into the coupling between flexoelectric polarization and photoexcited carriers. Despite the promising progress, significant challenges remain in quantitatively disentangling intrinsic flexoelectric contributions, implementing scalable and controllable strain engineering strategies, and ensuring mechanical robustness under operational conditions. Looking forward, we outline emerging pathways, including freestanding thin films, micro‐structured strain architectures, and multifunctional device integration, which harness flexoelectricity to enable adaptive, efficient, and mechanically responsive photoconversion systems for next‐generation energy and sensing technologies.

## Graphical abstract

Mechanical bending of a flexible cantilever induces a strain gradient in the photoactive material. The resulting flexoelectric field couples with photovoltaic and photoconductive effects, modulating charge generation, separation, and collection. A comparative analysis of oxide perovskites, halide perovskites, and two‐dimensional materials is presented, together with an evaluation of strain‐gradient loading methods.

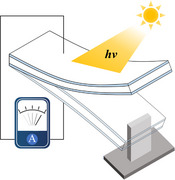

## 1 Introduction

### 1.1 Photoconversion Fundamentals

The quest to harness solar energy effectively hinges on our ability to seamlessly convert light into electricity, a technological feat made possible by advances in semiconductor physics. While the sun offers an abundant and clean energy source, the critical challenge lies not in the availability of the resource but in the efficiency and economy of its conversion. At the heart of this conversion process are solid‐state devices whose operation is governed by fundamental light–matter interactions. Two such cornerstone phenomena (the photovoltaic effect and photoconductivity) enable the detection [1, 2, 3, 4, 5, 6, 7, 8], photoluminescence [9, 10], and most importantly, the direct power generation from sunlight [11, 12, 13, 14, 15, 16, 17]. While often discussed in tandem, the photovoltaic effect and photoconductivity are distinct mechanisms with different operational principles and applications.

**FIGURE 1:**
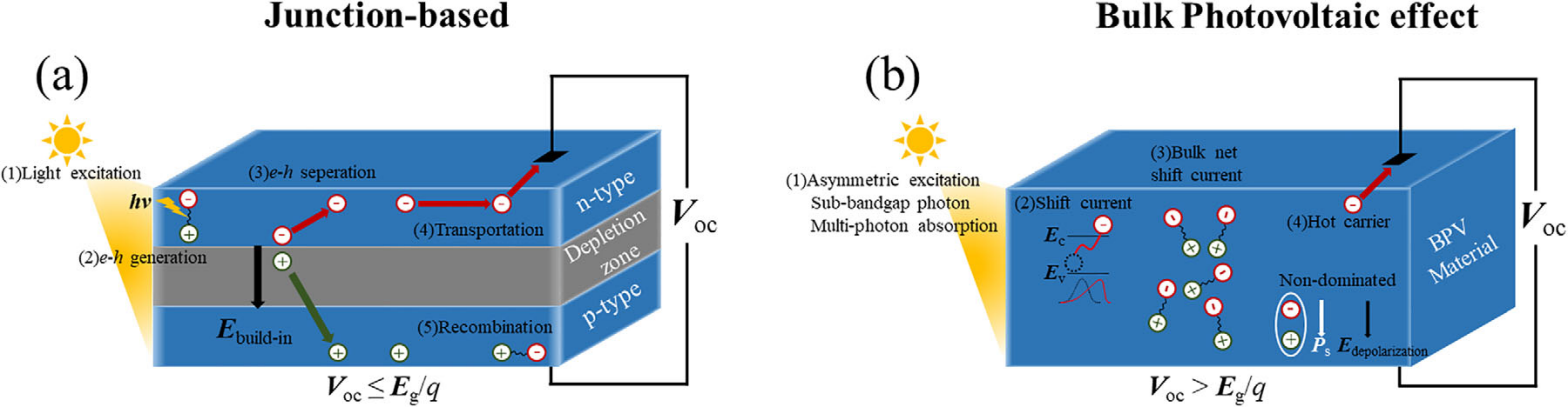
Mechanisms of (a) junction‐based and (b) bulk photovoltaic effects. Junction‐based photovoltaic devices are intrinsically limited to operation below the *S‐Q* efficiency limit and produce a *V*_oc_ that cannot exceed the material bandgap. In contrast, BPV devices, enabled by non‐centrosymmetric structures such as ferroelectrics, can surpass the *S‐Q* limit and generate a *V*_oc_ that exceeds the bandgap energy.

The photovoltaic (PV) effect is the physical process that forms the basis of solar cells. It involves the generation of a voltage and subsequently, an electric current, in a semiconductor material upon exposure to light. A critical distinction in the operational principles of photovoltaic devices lies in the origin of the photo‐induced charge separation, primarily categorized into the junction‐based [18, 19, 20, 21, 22] and the bulk photovoltaic effects (BPV) [23, 24, 25, 26, 27, 28], as shown in Figure 1. The former (Figure 1a), which underpins the vast majority of conventional solar cells, relies on a built‐in electric field created by structural asymmetry, such as a *p‐n* junction. This field efficiently separates electron‐hole pairs, leading to high photocurrents and mature, commercially viable technologies. In stark contrast, the BPV emerges in homogeneous, non‐centrosymmetric material systems without the need for junctions (Figure 1b). Its driving force is the crystal asymmetry itself, which causes asymmetric carrier scattering upon photoexcitation, generating a direct net current. A hallmark of the BPV is its potential to generate a photovoltage that can significantly exceed the material's bandgap, challenging the theoretical limits of conventional cells [29, 30, 31]. Despite the effort to improve its power output [29], a device based on the BPV effect comes with the trade‐off of relatively low photocurrent or photovoltage. While junction‐based mechanisms dominate current applications, the exploration of the BPV offers a promising pathway for next‐generation high‐voltage micro‐scale energy harvesters and novel optoelectronic devices, driven by fundamental quantum material properties [32, 33].

The mechanism of photovoltaic effects can be categorized into junction‐based and bulk photovoltaic phenomena. In a junction‐based device, as illustrated in Figure 1a, light absorption occurs within a functional layer whose bandgap matches the working spectrum. Photogenerated carriers are subsequently separated by the built‐in electric field formed at a semiconductor junction, such as a p‐n homojunction or a Schottky heterojunction. This conventional mechanism is fundamentally constrained by the Shockley‐Queisser (*S‐Q*) limit, with the achievable open‐circuit voltage *V*_oc_ remaining below the material's bandgap energy. In contrast, bulk photovoltaic materials, typically those lacking inversion symmetry such as ferroelectrics, can generate a photovoltage that exceeds the bandgap, as shown in Figure 1b. In these systems, carrier separation is driven by an internally spontaneous polarization field rather than a junction‐based field, enabling power conversion efficiencies (PCEs) that surpass the *S‐Q* limit.

Photoconductivity, in contrast, is a modulatory phenomenon. It describes the increase in electrical conductivity of a semiconductor or dielectric material when it absorbs light. The underlying mechanism similarly begins with the absorption of photons and the generation of electron‐hole pairs. However, in a simple photoconductor, there is no built‐in electric field to separate the charge carriers. Instead, the newly generated charge carriers increase the number of free electrons and holes available to conduct electricity, thereby reducing the material's electrical resistance. An external bias voltage must be applied to measure the change in conductivity. The resulting photocurrent is proportional to the intensity of the incident light, making photoconductors excellent in various photodetection applications, such as in photodetectors, light sensors, and Xerography [34, 35, 36, 37].

Building upon the foundational principles of the photovoltaic and photoconductive effects, the pursuit of higher energy conversion efficiency inevitably confronts its theoretical and practical boundaries. For example, in regard to conventional *p‐n* junction solar cells, the *S‐Q* limit establishes a fundamental ceiling, typically around 33.7% for a single junction under non‐concentrated sunlight [38]. This limit arises from two principal loss mechanisms: the inability to absorb photons with energy below the material's bandgap (transmission loss) and the thermalization of the excess energy from photons above the bandgap (thermalization loss). While strategies like forming multi‐junction or tandem solar cells can circumvent this limit, they could entail high complexity and cost [39, 40, 41, 42, 43, 44].

Encouragingly, the exploration of the BPV in non‐centrosymmetric materials, such as ferroelectrics, presents a tantalizing alternative. These materials leverage spontaneous polarization or the piezoelectric effect to generate a photovoltage that can, in theory, far exceed the bandgap, seemingly bypassing the *S‐Q* voltage constraint [30, 31]. However, this approach faces its own profound limitations. The very mechanism that enables the high photovoltage is often dependent on internal field‐induced charge separation across a narrow depletion region rather than a true bulk crystal effect, resulting in exceptionally low photocurrents by orders of magnitude compared to conventional cells [45, 46]. Furthermore, the wide bandgaps common to many strongly polar materials (e.g., BaTiO_3_) limit their absorption of the solar spectrum, and the stability and scalability of these systems remain significant challenges [47, 48]. Consequently, while BPV offers a fascinating physical phenomenon that defies traditional limits, its practical efficiency in energy harvesting has thus far been severely constrained, directing research toward engineering the effect in narrower‐gap materials or harnessing it in hybrid material systems and device architectures. While the limitations of *p‐n* junctions and traditional BPV effects are evident, the emerging role of flexoelectricity presents a novel and promising pathway for enhancing light‐energy conversion, further advancing energy‐related and optoelectronic device applications.

### 1.2 Fundamentals of Flexoelectricity

Piezoelectricity, which arises from the non‐centrosymmetric crystal structure, has long been exploited for electromechanical energy conversion in various applications. In recent years, the concept of piezoelectricity has been further extended to a family of piezo‐related effects, including piezotronics, piezophototronics, piezocatalysis, and piezoresistivity, where strain‐induced polarization plays a central role in modulating electronic, optoelectronic, chemical, and transport properties of materials [49, 50, 51, 52]. Notably, a growing body of evidence across these fields has revealed that the conventional picture based solely on uniform lattice deformation is insufficient, particularly at the nanoscale. Instead, strain gradients ‐can give rise to a distinct phenomenon known as flexoelectricity, which refers to the polarization induced by a gradient of strain (or inhomogeneous deformation). Unlike piezoelectricity, flexoelectricity is a universal effect present in all dielectrics regardless of crystal symmetry, and it becomes increasingly significant as the feature size decreases. Therefore, understanding flexoelectricity is essential for capturing the full electromechanical response in nanoscale systems, bridging the gap between classical piezoelectric theories and emerging strain‐gradient‐enabled functionalities.

The flexoelectric effect, a phenomenon where a strain gradient induces an electrical polarization in any dielectric material, has a fascinating history that spans over half a century. Its story begins not with a bang, but with a subtle theoretical prediction. The concept was first explicitly theorized in the late 1950s and early 1960s by scientists like Kogan, Mashkevich, and Tolpygo in the Soviet Union [53]. For decades, it remained a scientific curiosity, which has, however, been largely overshadowed by its close relative, the piezoelectric effect. The reason was simple: in most bulk materials, the flexoelectric effect is exceptionally weak. The polarization it generates was considered negligible compared to piezoelectricity, which requires non‐centrosymmetric crystals and is driven by uniform strain. Consequently, research throughout the 20th century was confined to a niche area of fundamental physics, with limited practical application. The turn of the 21st century marked a dramatic turning point, driven by two interconnected technological revolutions: the ascent of nanoscience and the refinement of epitaxial growth techniques. It was realized that the strength of the flexoelectric effect is inversely proportional to the size of the material. As structures shrunk to the nanoscale, strain gradients could become enormous, making the flexoelectric effect not just significant, but often dominant [54, 55, 56, 57, 58].

**FIGURE 2:**
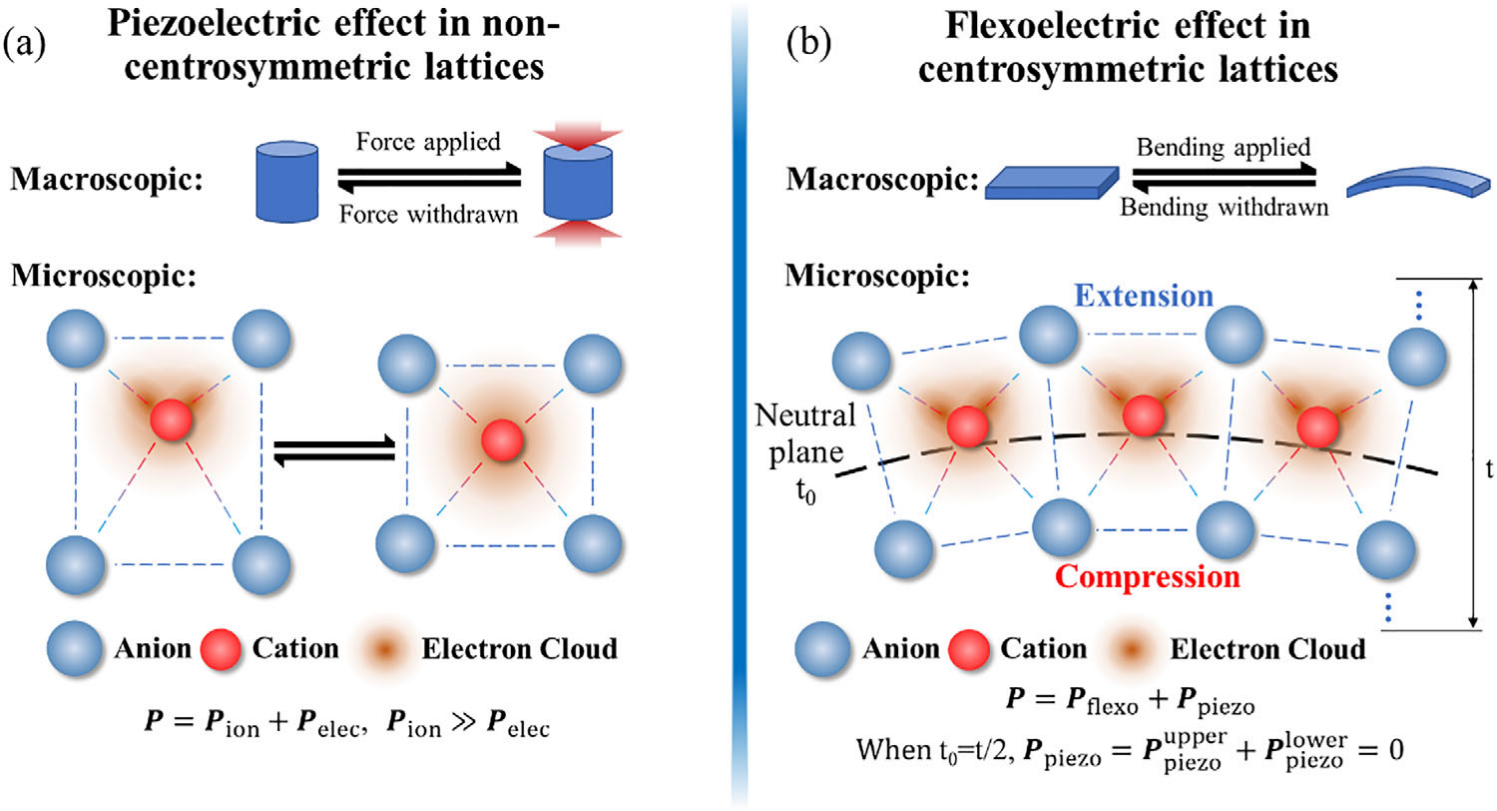
Mechanisms of (a) piezoelectric effect in non‐centrosymmetric lattices and (b) flexoelectric effect in centrosymmetric lattices. The piezoelectric effect is restricted to non‐centrosymmetric crystal structures, as it relies on intrinsic symmetry breaking to couple mechanical strain and electric polarization. In contrast, the flexoelectric effect arises universally in all dielectric materials, since a strain gradient (whether induced by bending, indentation, or heterogeneous deformation) locally breaks inversion symmetry, thereby generating electric polarization regardless of the material's bulk crystal symmetry.

The piezoelectric effect refers to the phenomenon where a dielectric material develops an electric polarization under applied mechanical stress, as demonstrated in Figure 2a. Because the polarization is strongly coupled to lattice distortion through the material's elastic energy, the application of an external electric field also induces mechanical strain, a phenomenon known as the converse piezoelectric effect. The mathematical expressions for these two effects are given by [59]:

$$\;P_{i}=d_{ijk}\;T_{jk}\;\left(\mathrm{Direct}\;\mathrm{piezoelectric}\;\mathrm{effect}\right)$$

$$\;S_{jk}=d_{ijk}\;E_{i}\left(\mathrm{Converse}\;\mathrm{piezoelectric}\;\mathrm{effect}\right)$$

where *P_i_* represents the polarization component along the *i* direction, *d*_*ijk*_ is the piezoelectric coefficient tensor, *T_jk_* is the stress component, *S_jk_* is the strain component, and *E_i_* represents the electric field component toward the *i* direction. The direct piezoelectric relation indicates that the induced polarization in a piezoelectric material is proportional to both its piezoelectric coefficient and the magnitude of the applied stress. Conversely, the constitutive equation for the converse piezoelectric effect shows that the resulting strain is proportional to the material's piezoelectric coefficient and the strength of the applied electric field.

In a crystalline lattice, the total polarization arises from two distinct microscopic mechanisms: ionic polarization due to relative displacements of charged ions, and electronic polarization due to distortion of the electron cloud. The total, ionic, and electronic polarization can be expressed as:

$$P_{\mathrm{ion}}=\frac{1}{V}\;\sum_{i}Z_{i}^{\mathrm{ion}}ed_{i}$$

$$\;P_{\mathrm{ion}}=\frac{1}{V}\;\sum_{i}Z_{i}^{\mathrm{ion}}ed_{i}$$

$$P_{\mathrm{elec}}=-\frac{2e}{{\left(2\pi \right)}^{3}}\int_{\mathrm{BZ}}^{.}\langle u_{nk}|i\nabla_{k}|u_{nk}\rangle dk$$

where *V* is the cell volume, *Z_i_* ^ion^ *e* is the ionic charge, and *d_i_* is the displacement of ion *i*. The electronic contribution is integrated over the Brillouin zone, with *u_n_*_k_ being the periodic part of the Bloch wavefunction.

The flexoelectric effect describes the coupling between electric polarization and strain gradient in a material, as shown in Figure 2b. Analogous to the piezoelectric effect, the flexoelectric effect also possesses a converse form, termed the converse flexoelectric effect, which captures the coupling between stress and electric field gradient. The mathematical expressions for the direct and converse flexoelectric effects are given by [60, 61]:

$$\;P_{i}=\mu_{ijkl}\;\frac{\partial S_{jk}}{\partial x_{l}}\;\left(\mathrm{Direct}\;\mathrm{flexoelectric}\;\mathrm{effect}\right)$$

$$\;T_{ij}=\mu_{ijkl}\;\frac{\partial E_{l}}{\partial x_{k}}\left(\mathrm{Converse}\;\mathrm{flexoelectric}\;\mathrm{effect}\right)$$

where *T_ij_* is the stress component, *µ_ijkl_* denotes the flexoelectric coefficient tensor, $\frac{\partial S_{jk}}{\partial x_{l}}$ is the gradient of the strain component *S_kl_* along the *x_j_* direction, and $\;\frac{\partial E_{l}}{\partial x_{k}}$ represents the gradient of the electric field component *E_l_* along the *x_k_* direction. These constitutive relations indicate that the induced polarization is directly proportional to both the flexoelectric coefficient and the applied strain gradient. If the neutral bending plane is located exactly at the mid‑thickness of a piezoelectric sheet, the upper half (under tension) and the lower half (under compression) develop opposite polarizations due to the converse piezoelectric effect.

When it comes to light energy conversion, the flexoelectric effect offers distinct advantages over its predecessors, regarding junctions and BPV. First, it is a universal property present in all dielectric solids, irrespective of their crystal symmetry. This dramatically expands the material palette beyond centrosymmetric restrictions of ferroelectrics, allowing the utilization of high‐performance, common semiconductors like silicon or perovskites that were previously limited to junction‐based mechanisms [62, 63, 64]. Second, the magnitude of the induced polarization and the resulting photo‐voltage is gradient‐tunable. Unlike the fixed built‐in field of a *p‐n* junction or the spontaneous polarization of a ferroelectric, the flexoelectric field can be exquisitely controlled by engineering the strain gradient profile, offering a powerful knob to optimize charge separation without altering the base material's composition [65, 66, 67, 68]. Crucially, in semiconducting nanostructures (e.g., nanowires, bent membranes) where large strain gradients naturally occur, the flexoelectric effect can generate substantial built‐in fields comparable to or even exceeding those in the *p–n* junctions [69, 70, 71]. This enables highly efficient, directional separation of photogenerated charge carriers within a single material, mitigating the fundamental current‐voltage trade‐off by reducing charge recombination. Furthermore, the synergy between flexoelectricity and other BPV mechanisms in suitable materials can lead to a cumulative or even enhanced photovoltaic response. Therefore, flexoelectricity transcends the traditional dichotomy, enabling the design of novel, gradient‐engineered photovoltaic or optoelectronic devices that combine the material versatility of conventional semiconductors with the high‐voltage potential of bulk effects.

### 1.3 Coupling of Flexoelectricity and Photovoltaics

The flexoelectric effect, which generates an effective internal electric field *E*_flexo_ in response to a strain gradient, exhibits profound bidirectional coupling with light‐matter interactions, particularly the photovoltaic effect and photoconductivity. These couplings create positive feedback loops that significantly enhance device performance, while inevitably introducing ambiguity in the distinction between intrinsic and effective flexoelectric effect.

A significant and persistent challenge in flexoelectricity research is the considerable discrepancy between theoretically predicted intrinsic flexoelectric coefficients and those measured experimentally. Theoretical models often calculate the response of a perfect, insulating lattice to a strain gradient [72, 73]. However, in lab‐based experiments, the measured “effective” flexoelectric response could be a superposition of the intrinsic effect and extrinsic contributions from various charged microstructures. These extrinsic sources mainly include mobile charge carriers [74, 75, 76, 77, 78, 79], surface states [77, 80, 81, 82, 83, 84], defects [85, 86], and domain structures [87, 88, 89]. While this complexity makes it exceptionally difficult to isolate and measure the intrinsic flexoelectric effect in experiments, it simultaneously presents a remarkable opportunity for practical applications. The very fact that these extrinsic factors can amplify the overall response performance provides a viable pathway for engineering materials with a giant effective flexoelectric coefficient [74, 76, 77, 78, 81, 83, 86, 90].

Among the various strategies, charge carrier doping has proven to be a particularly powerful and versatile approach. A substantial body of research has demonstrated that introducing additional charge carriers, regardless of their origin, can lead to a dramatic enhancement of the experimentally measured effective flexoelectric effect [74, 75, 76, 77, 78, 79]. The underlying mechanism is twofold. First, the increased carrier density provides more charges to screen the flexoelectric polarization. This more effective screening leads to a larger measurable flexoelectric‐induced polarization and, consequently, a larger voltage or current in a direct flexoelectric measurement. Second, the flexoelectric‐induced carrier transport plays an important role; that is, the flexoelectric field itself can drive the directional movement of these free carriers, generating an effective current that directly adds to the intrinsic flexoelectric current in the typical flexoelectric measurements.

Illumination provides the charges necessary to manifest the flexoelectric effect in dynamic measurements. When a material is subjected to bending or vibrational deformation, the associated strain gradient moves in time, and the *E*_flexo_ field attempts to redistribute charges to screen itself. Under dark conditions, the density of available free charge carriers may often be limited. In contrast, under illumination, photogenerated carriers provide a plentiful source of charge carriers. These carriers are rapidly mobilized by the dynamic *E*_flexo_ field, leading to a measurable alternating or pulsed current. Consequently, both the screening charge and the collected current induced by mechanical excitations like bending vibrations are dramatically amplified under light. This principle holds true whether the carriers are introduced through doping [74, 75, 76, 77, 79] or generated transiently through photoexcitation [76, 78]. In both cases, the resulting influx of mobile charges significantly boosts the macroscopic output, turning the challenge of extrinsic interference into a design principle for creating high‐performance flexoelectric materials and devices, including sensors, energy harvesters, and nano‐actuators.

In the reverse direction, under uniform illumination, the BPV effect generates a photocurrent or photovoltage in non‐centrosymmetric materials (like ferroelectrics) or even in centrosymmetric materials subjected to strain gradients [23, 24, 25, 26, 27, 28]. The flexoelectric effect powerfully complements this process. The strain‐gradient‐induced *E*_flexo_ acts as a built‐in, spatially varying electric field that actively facilitates the separation of photogenerated electron–hole pairs. This field prevents their recombination, efficiently sweeping electrons and holes in opposite directions. This process directly leads to a substantial enhancement of both the open‐circuit voltage *V*_oc_ and the short‐circuit current *J*_sc_, far exceeding what either effect could achieve independently. This coupling describes a synergistic relationship in which the flexoelectric effect enhances the BPV effects and, conversely, photovoltaic processes reinforce flexoelectric functionality.

As for the coupling between the flexoelectric effect and photoconductivity, the primary link is mediated through localized field enhancement. Strain gradients are naturally concentrated at microstructural features such as grain boundaries, defects, and domain walls, due to sudden changes in lattice structure [85, 86, 91, 92]. At these specific locations, the flexoelectric effect generates the strong, localized *E*_flexo_ and corresponding redistribution of charge carriers, leading to elevated local charge densities and band bending. Under illumination, these localized fields play two key roles. First, they enhance the separation of photogenerated charge carriers, similar to the photovoltaic case but focused on specific microstructural features. Second, they can locally lower the energy barriers for charge carrier transport (e.g., by reducing or flattening band bending at a grain boundaries) [91, 92]. The combined effect of increased free carrier densities and their facilitated transport leads to a significant enhancement of the photoconductive gain, enabling a much larger increase in electrical conductivity for a given light intensity than would be possible in the absence of the flexoelectric activity.

The reverse coupling is equally critical. The photoconductivity induced by light illumination determines how efficiently the material can supply the charge carriers required to screen a flexoelectric field. Higher photoconductivity enhances the flexoelectric effect primarily by providing free carriers that screen the internal electric field generated by a strain gradient. When the material exhibits high photoconductivity, photoexcited electrons and holes can quickly redistribute to partially or fully screen this internal field. This screening reduces the opposing electrostatic force that normally limits further polarization or strain gradient, effectively “unpinning” the flexoelectric response. Consequently, the material can sustain a larger strain gradient under the same external stimulus, leading to a significantly enhanced effective flexoelectric coefficient or output signal [76, 77].

In summary, these bidirectional couplings reveal a powerful synergy in which mechanical strain gradients and light illumination mutually amplify each other's electronic responses. This interplay opens new avenues for the development of high‐performance mechanical energy harvesters, ultrasensitive photodetectors, and adaptive optoelectronic systems.

### 1.4 Scope and Roadmap

A review dedicated to the coupling between flexoelectricity and photoconversion is of critical importance for advancing modern materials science and nanoscale device technologies, particularly in the fields of energy conversion and optoelectronics. Despite rapidly growing experimental and theoretical interest, a comprehensive and unified overview of this emerging interplay is still lacking. This review is therefore intended to serve as a timely and essential catalyst, not merely by consolidating existing knowledge, but by articulating a new paradigm for actively controlling material properties through the synergistic interaction of mechanical strain gradients and light.

The significance of this review is threefold, with broad and lasting implications. First, at the fundamental level, it provides a coherent framework for understanding how flexoelectric fields and photoexcited charge carriers interact across multiple length scales, from atomic defects to mesoscale microstructures. Second, from a mechanistic perspective, it clarifies the reciprocal coupling pathways that govern charge separation, transport, screening, and recombination under simultaneous mechanical and optical stimuli. Third, in terms of practical impact, it offers concrete design principles for next‐generation energy, optoelectronic, and electronic devices that exploit flexo‐photo coupling to achieve enhanced efficiency, sensitivity, and multifunctionality.

**FIGURE 3:**
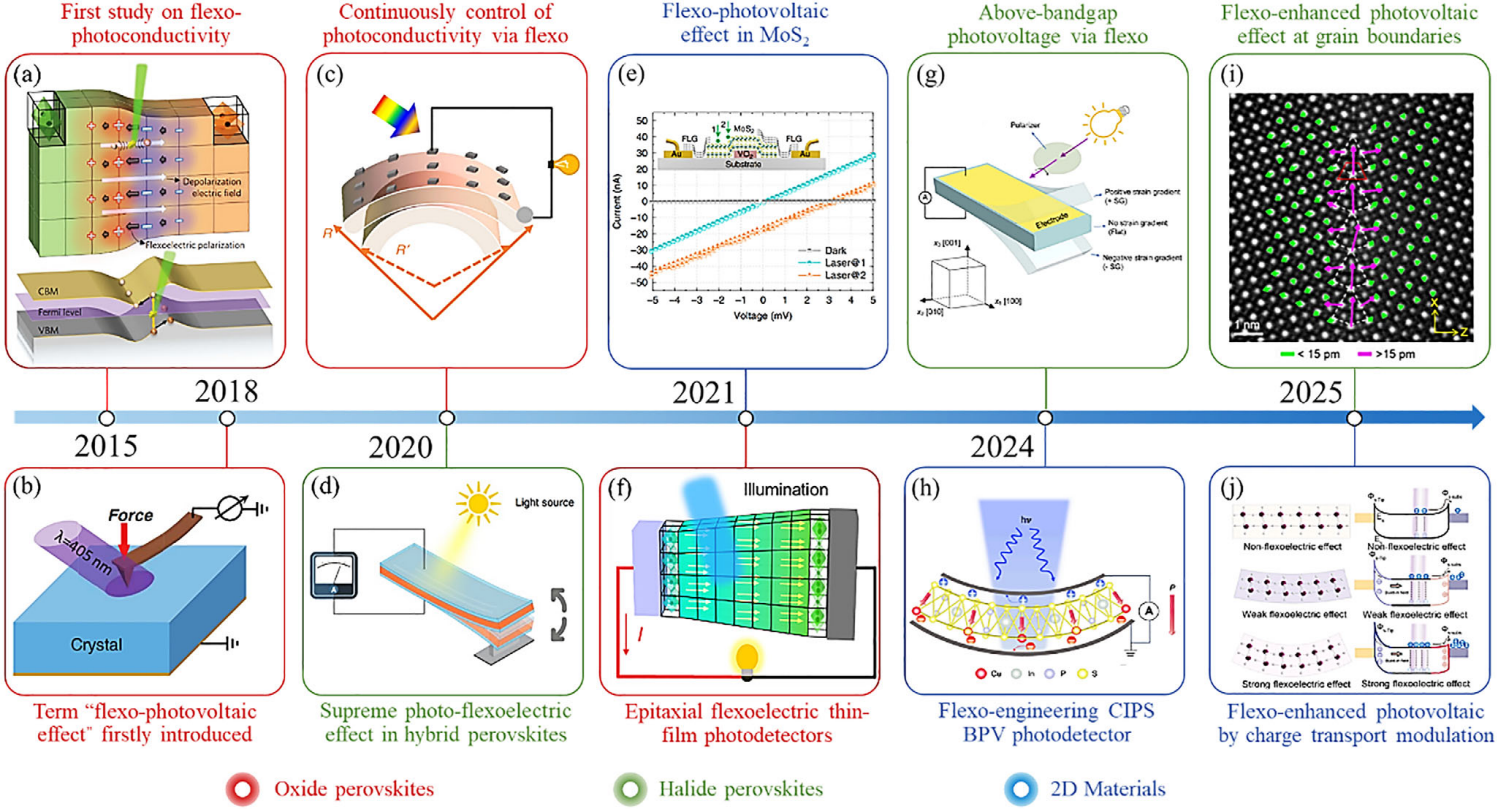
History of flexo‐photoconversion research based on three types of materials. (a) The first study revealed enhanced photoconductivity in BiFeO_3_ charged domain walls due to the flexoelectric effect. Adapted with permission from ref. [93] Copyright 2015 Springer Nature Limited. (b) AFM probes induced flexo‐photovoltaic effect in centrosymmetric oxides. Adapted with permission from ref. [94] Copyright 2018 The Authors, some rights reserved; exclusive licensee American Association for the Advancement of Science. (c) Continuous control of photoconductivity in freestanding BiFeO_3_ film via tunable bending. Adapted with permission from ref. [95] Copyright 2020, CC BY 4.0. (d) Supreme photo‐flexoelectric effect in hybrid halide perovskites under illumination. Adapted with permission from ref. [78] Copyright 2020 Springer Nature Limited. (e) Flexo‐photovoltaic effect in 2D MoS_2_ via the deformation of phase transition material VO_2_. Adapted with permission from ref. [96] Copyright 2021 Springer Nature Limited. (f) Photodetectors based on epitaxial‐mismatch‐induced flexoelectric effect in LaFeO_3_/LaAlO_3_ system. Adapted with permission from ref. [97] Copyright 2021 American Chemical Society. (g) Flexo‐photovoltaic effect and above‐band‐gap photovoltage induced by flexoelectric effect in MAPbI_3_ halide perovskite. Adapted with permission from ref. [98] Copyright 2024 American Physical Society. (h) Flexoelectric engineering of BPV photodetector in 2D CuInP_2_S_6_. Adapted with permission from ref. [99] Copyright 2024 American Chemical Society. (i) Enhanced photovoltaic effect at the grain boundaries of CsPbBr_2_Cl and CsPbBr_3_. Adapted with permission from ref. [92] Copyright 2025 American Chemical Society. (j) Flexoelectric enhanced photovoltaic effect by charge transport modulation and band engineering in 2D α‐MoO_3_. Adapted with permission from ref. [100] Copyright 2025 Elsevier Ltd.

In this review, we systematically delve into the emerging role of flexoelectricity in modulating photovoltaic and photoconductive phenomena, moving beyond fundamental principles to explore experimental realizations and their profound implications across three distinct yet pivotal material platforms: oxide perovskites, halide perovskites, and two‐dimensional (2D) Van der Waals materials, as shown in Figure 3. Before we systematically review these three classes of material systems in the following sections, we could like to provide a concise overview here.

For a long time, flexoelectric and photoelectric effects were investigated as two independent and non‐overlapping fields. Several reasons account for this separation. First, the large dimensions and poor growth quality of materials led to insignificant flexoelectric responses. Second, appropriate methods for applying strain gradients were lacking. Third, the understanding of materials that exhibit both excellent flexoelectric and photoelectric properties was still evolving. With improvements in material fabrication and the diversification of strain‐gradient application techniques, studies on the coupling between flexoelectric and photoelectric effects first emerged in inorganic perovskites. Under various experimental conditions, synergistic enhancement of both effects was observed to varying degrees. Subsequently, as hybrid perovskites became widely studied, the two effects exhibited more pronounced synergistic enhancement, steering the research focus toward solar cell applications. More recently, in emerging 2D materials, research on flexoelectric and photoelectric effects has progressed in parallel, naturally leading to investigations of their coupling.

From a chronological perspective, the first studies explicitly proposing the coupling of these two effects appeared around 2015, meaning this field is just over a decade old. In terms of material systems, there has been a shift from conventional inorganic perovskites toward hybrid perovskites and 2D materials. Meanwhile, research content has evolved from fundamental mechanistic studies toward practical applications. By progressing from correlated oxides to soft ionic semiconductors and ultimately to atomically thin 2D systems, this review aims to consolidate current understanding and inspire future research into strain‐gradient engineering as a universal strategy for overcoming efficiency bottlenecks and enabling novel, flexible energy‐related and optoelectronic devices.

## 2 Flexo‐Photoconversion in Oxide Perovskites

### 2.1 Material Systems and Their Features

The story of traditional oxide perovskites can be traced back to 1839, when the German mineralogist Gustav Rose identified a calcium titanium oxide mineral (CaTiO_3_) in the Ural Mountains and named it “perovskite” in honor of the Russian mineralogist Lev Perovski. For over a century, perovskite remained primarily a mineralogical curiosity. A significant turning point emerged in the mid‐20th century, when scientists realized that the perovskite crystal structure serves as a common framework for a vast family of compounds exhibiting immensely diverse and useful physical properties. This realization sparked intense research into synthetic oxide perovskites, leading to seminal discoveries such as ferroelectricity in barium titanate (BaTiO_3_) in the 1940s [101, 102], which became foundational for capacitor technologies, and the breakthrough discovery of high‐temperature superconductivity in copper‐oxide‐based perovskites in 1986. The latter achievement earned Bednorz and Müller the Nobel Prize [103]. These established the perovskite family as a cornerstone of modern solid‐state physics and materials science.

**FIGURE 4:**
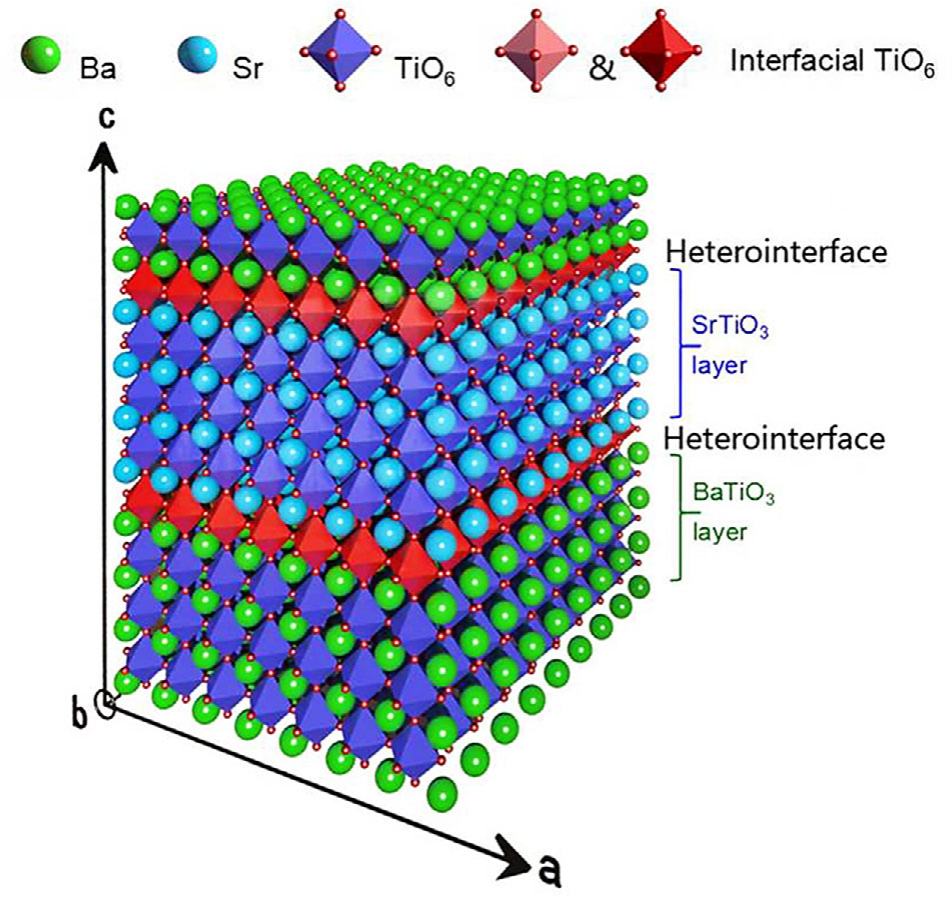
Schematic illustrations of BTO/STO/BTO oxide perovskite heterostructure. Adapted with permission from ref. [104] Copyright 2015 American Chemical Society.

Despite the immense chemical diversity across this family, traditional oxide perovskites share a common defining feature: their crystal structure. The ideal structure is described by the general formula *A*BO_3_, in which a larger ‘*A*’ cation (e.g., La^3+^, Sr^2+^, Ca^2+^) occupies the cube corners with 12‐fold coordination, while a smaller ‘*B*’ cation (e.g., Ti^4+^, Mn^3+^, Fe^3+^) resides at the body center with sixfold coordination, forming a framework of corner‐sharing *B*O_6_ octahedra [104], as shown in Figure 4. Fundamental physical properties in perovskite materials often arise from the underlying lattice framework. For instance, the ferroelectricity in BTO primarily stems from the displacement of the Ti^4+^ ion away from the center of its TiO_6_ octahedral coordination [105]. This mechanism of symmetry breaking via cation off‐centering can also extend to other perovskite systems, including metal‐free halide perovskites, which will be discussed in the following section. This seemingly simple architecture is remarkably flexible and often distorted, giving rise to a host of crucial physical properties. These include ferroelectricity [104, 105, 106], piezoelectricity [107, 108, 109], and colossal magnetoresistance [110, 111], and a rich spectrum of electronic behaviors spanning insulating, metallic [112, 113, 114], and superconducting states [115, 116, 117]. Furthermore, their ionic conductivity makes them excellent electrolytes in solid oxide fuel cells, while their catalytic activity is harnessed in applications like automotive exhaust catalysts [118, 119, 120].

The exploration of the flexoelectric effect in oxide perovskites began as a scientific curiosity, initially considered a weak phenomenon relevant only to niche applications. Early theoretical work in the mid‐20th century established that a strain gradient could induce polarization in any dielectric material; however, experimental verification was limited, and the measured coefficients were small [53]. The field witnessed a paradigm shift in the early 2000s with the discovery that the flexoelectric effect is dramatically enhanced in ferroelectric perovskites, particularly in the ferroelectric phase of barium titanate (BaTiO_3_, BTO) [121]. Researchers found that the flexoelectric coefficient in BTO is orders of magnitude larger than classic theoretical predictions, primarily due to its strong coupling with the material's high dielectric constant and intrinsic polarization. This breakthrough established ferroelectric perovskites as the premier materials for studying giant flexoelectricity. The quest for even larger coefficients later identified strontium titanate (SrTiO_3_, STO) as another champion material [122]. Despite being a quantum paraelectric, STO exhibits an exceptionally large flexoelectric response at low temperatures, attributed to its very high dielectric constant and lattice softness, which allows large strain gradients to be efficiently converted into polarization. Building on the discoveries of giant flexoelectricity in BTO near its ferroelectric Curie point and in STO at cryogenic temperatures, research logically progressed to their solid solutions, barium strontium titanate (Ba_x_Sr_1‐__x_TiO_3_, BST). A series of studies in the 2000s experimentally confirmed that, with an optimal compositional tuning, BST could exhibit a substantially larger flexoelectric coefficient at room temperature than either of its end‐member compounds [123, 124]. This enhancement stems from the “critical point” behavior in the BST phase diagram. When the composition (typically in the range of *x* = 0.5‐0.7) is tuned such that the material lies near the boundary between the paraelectric and ferroelectric phases at room temperature (i.e., in a relaxor‐like ferroelectric state), the dielectric constant reaches a broad maximum. Because the flexoelectric coefficient is positively correlated with the dielectric permittivity (*µ*_*ij*_ = *f*_*ijkl*_ × *ε*_*kl*_, where *f* is the flexo‐coupling coefficient, usually considered as constant for a certain material), this exceptionally high dielectric response makes the material extremely sensitive to external strain gradients, efficiently converting minute deformations into macroscopic polarization. Studies on compositions like Ba_0.67_Sr_0.33_TiO_3_ have demonstrated room‐temperature flexoelectric coefficients several times larger than those of pure BTO or STO [123, 124]. This finding carries significant practical implications, as it proves that superior flexoelectric performance could be achieved at room temperature without relying on complex and costly cooling systems, thereby opening a viable pathway toward practical flexoelectric devices, including mechanical sensors, energy harvesters, and novel electronic components.

Accurately measuring the flexoelectric coefficient *µ* has remained a central challenge and a key driver of methodological innovation in the field. The most widely used methods involve generating a controlled, uniform strain gradient and measuring the resulting electrical response. A classical configuration is the cantilever bending experiment, in which a thin perovskite beam is clamped at one end and mechanically deflected at the other, generating a linear strain gradient across its thickness [123, 125]. The induced voltage difference between the top and bottom surfaces is then measured under open‐circuit conditions, or the generated charge is detected under short‐circuit conditions, enabling extraction of the *µ*. To overcome complications associated with clamping effects and to probe flexoelectricity at smaller scales, researchers have developed more sophisticated techniques. These include using atomic force microscopy (AFM) with a conductive tip to indent the material's surface, creating a highly localized strain gradient and directly measuring the generated flexoelectric voltage or current. This method is particularly useful for characterizing thin films and mapping the flexoelectric response at the nanoscale [94, 126, 127].

Traditional oxide perovskites such as BiFeO_3_ (BFO) and BTO exhibit photovoltaic effects that are primarily driven by their ferroelectric polarization. The polarization creates a strong built‐in electric field capable of separating photogenerated charge carriers. However, their photovoltaic performance in single‐junction solar cells is limited by their relatively wide bandgaps, typically exceeding 2.7 eV for BFO and around 3.2 eV for BTO, which restricts their absorption of visible light [128, 129]. Consequently, although these materials can achieve respectable open‐circuit voltage *V*_oc_, for instance, BFO‐based thin films have exhibited *V*_oc_ values of 0.3–0.4 V, their short‐circuit current densities *J*_sc_ are generally very low, often below 1 mA/cm^2^, due to the limited photons they can absorb. This fundamental trade‐off between voltage and current results in low PCEs, which are typically reported to be below 2% for these materials [130].

Within the family of oxide perovskites, BFO‐based materials are currently the most promising for photovoltaic applications from the perspective of bandgap tunability and conversion efficiency. Although still wide, the bandgap of BFO is more amenable to modification than that of pure BTO. Research has shown that elemental doping, particularly with lanthanum (La), can reduce BFO's bandgap to around 2.71 eV and further optimize its ferroelectric properties [131, 132, 133], leading to enhanced *V*_oc_. Recent studies on optimized, phase‐pure La‐doped BFO films have reported enhanced performance, with PCEs reaching approximately 1.9% [132]. While this efficiency is modest compared to halide perovskites or silicon, it highlights BFO's potential as a lead‐free and stable photoactive medium. The primary value of these materials may not lie in direct competition with conventional solar cells, but rather in niche applications that leverage their unique ferroelectric photovoltaic properties, such as optoelectronic memory devices, or in tandem structures where their wide bandgap could be complementary to narrower‐gap absorbers.

Building on the understanding of their inherent photovoltaic limitations, such as wide bandgaps and modest carrier separation efficiencies, recent research has unveiled a promising pathway to enhance the performance of oxide perovskites like BFO: the strategic coupling of flexoelectricity with the photoconversion process. This approach leverages the fact that strain gradients and the flexoelectric effect can not only generate a giant polarization field to better separate photogenerated electron–hole pairs but can also actively modify the local band structure. Therefore, by intentionally engineering strain gradients into these materials, it is possible to create a synergistic “flexo‐photovoltaic” effect that simultaneously boosts the open‐circuit voltage and increases the photocurrent generation, offering a novel pathway to overcome the traditional performance limitations.

### 2.2 Progress, Strategies, and Mechanisms

We further classify the works based on the strategies employed to introduce strain gradients or flexoelectric effects. The strategies include: (a) AFM‐probe‐induced local strain gradients; (b) Macroscopic bending of thin films or devices, for example, cantilever bending, three‐point bending, and so on; (c) spontaneously formed strain gradients, such as lattice‐mismatch relaxation or composition gradients, domain patterns, grain boundaries, or defects. The magnitude, repeatability, and scalability of strain gradient induction methods are listed in Table 1.

**TABLE 1:** Magnitude, repeatability, and scalability of various strain gradient induction methods in oxide perovskite materials.

Source of strain gradient	Magnitude (m^−1^)	Repeatability	Scalability
AFM probe pressing	10^7^	Low	Very low
Cantilever bending	10^−2^–10^0^	High	Moderate
Uniform bending	10^0^–10^5^(freestanding)	High	Moderate
Substrate mismatch	10^6^–10^7^	Low–moderate	High
Domain walls	10^7^	Low	High

For oxide perovskite materials, macroscopic cantilever bending and three‐point bending offer high repeatability and well‐defined global strain gradients, making them suitable for quantitative flexoelectric studies, albeit with moderate scalability. In contrast, substrate‐mismatch strain relaxation and ferroelastic domain walls provide excellent scalability and built‑in gradients across wafer‑scale samples, yet suffer from lower repeatability due to their stochastic or history‐dependent distribution. AFM tip loading remains limited to local probing with poor reproducibility and scalability, primarily suited for microscopic domain manipulation rather than controlled strain gradient engineering. More specific strengths and drawbacks are listed in Table 2, and Table 3 lists the summary of relevant studies.

**TABLE 2:** Strengths and drawbacks of various strain gradient induction methods in oxide perovskite materials.

Source of strain gradient	Strengths	Drawbacks
AFM probe pressing	Large localized strain gradient in situ local measurement	Downward strain gradient only only applied to the surface area
Cantilever bending	Suitable for dynamic test simple simulation model	Small strain gradient flexible samples required
Uniform bending, for example, three‐point bending	Large strain gradient achievable on freestanding systems	Potential friction or defects on apply area
Substrate mismatch	Large biaxial strain gradient tunable by thickness and composition	Not actively controllable limitation on material selection
Domain walls	Large localized strain gradient multi‐field control available	Confined to the domain walls rely on the domain structures

**TABLE 3:** Summary of flexo‐photoconversion studies in oxide perovskites.

Source and magnitude of strain gradient	Material system	Properties/phenomenon	Ref
AFM probe pressing ∼10^7^ m^−1^	STO, TiO_2_, p‐Si single crystal	*I*_sc_ measured by conductive probe enhanced by 10^3^ for STO and TiO_2_, 10^2^ for p‐Si	[94]
**Macroscopic bending**			
Uniform curvature bending ∼10^5^ m^−1^	Freestanding BFO/LSMO	*V*_oc_ and *J*_sc_ are capable of continuous enhancement up to 24% and 90%, respectively.	[95]
Single‐clamped cantilever bending ∼10^0^ m^−1^	STO_‐δ_ single crystal	Flexoelectric effect in STO_‐δ_ is enhanced by more than 10^1^ under UV illumination	[76]
Single‐clamped cantilever bending ∼0.05 m^−1^	KNN/PVDF‐TrFE	Photovoltaic current *I*_pv_ enhanced by 35% compared with the flat state	[134]
Single‐clamped cantilever vibration	ITO/BFO/LNO/Mica	Charge, current, voltage, and power are enhanced under simultaneous light and vibration compared to under only light or vibration	[90]
In single‐clamped cantilever bending, a strain gradient is formed between the BTO nanoparticles	BTO/PVDF	Flexoelectric coefficients and photovoltaic current *I*_pv were_ enhanced by 3.7‐fold and 3.4‐fold, respectively	[135]
Uniform curvature bending ∼35 m^−1^	PZT nanowires/PDMS	Photovoltaic current *I*_pv_ increased by 27.8% comparing to the flat state	[136]
Uniform curvature bending ∼0.25 m^−1^	NNO nanotubes/Epoxy	Photovoltaic current *I*_pv_ increased by 79.9% comparing to the flat state	[137]
Single‐clamped cantilever vibration	ITO/BFO/LNO/Mica	Charge, current, voltage, and power enhanced under simultaneous light and vibration from room temperature to 130°C, compared to under only light or vibration	[138]
Uniform curvature bending ∼10^5^ m^−1^	LFO/LNO/buffer/Mica	*J*_sc_ capable of modulation from −40% to 20% by bending upward and downward	[66]
**Spontaneously formed strain gradients**			
Mismatch strain relaxation and gradient doping 0.32 × 10^5^ m^−1^	La, Co‐doped BFO	*J*_sc_ enhanced 2.7‐fold compared to the control composition	[139]
Mismatch strain relaxation and gradient doping ∼0.21 × 10^5^ m^−1^	Sm, Ni‐doped BFO	*J*_sc_ and *V*_oc_ enhanced 2.7‐fold and 9.8‐fold compared to the control composition, respectively	[140]
Mismatch strain relaxation and gradient doping	Pr, Co‐doped BFO	*J*_sc_ was enhanced 10‐fold compared to the control composition	[141]
Mismatch strain relaxation and changing temperature ∼10^7^ m^−1^	PZT/SRO/STO	*V*_oc_ increased by 0.5 V through enhancing the strain gradient	[142]
Mismatch strain relaxation ∼10^6^ m^−1^	LFO/LAO	*J*_sc_ of 0.21 mA/cm^2^ and *V*_oc_ of −0.2 V induced by the flexoelectric effect	[97]
Mismatch strain relaxation ∼10^6^ m^−1^	BNN doped BTO/SRO/STO	Nonswitchable *J*_sc_ of 11.2 µA/cm^2^ and *V*_oc_ of 0.3 V induced by the flexoelectric effect	[143]
Surface strain relaxation induced by polishing	BNT and BNT‐BFO ceramics	*I*_sc_ of −0.11 nA and *V*_oc_ of 7.3 V obtained through flexoelectric‐induced surface APV effect in paraelectric BNT‐BFO	[144]
Morphotropic phase boundaries ∼3.3 × 10^7^ m^−1^	BFO/LAO	Photocurrent in DWs was enhanced by over two orders of magnitude	[93]
Morphotropic phase boundaries ∼8 × 10^7^ m^−1^	BFO/LAO	The conductivity of the R phase is enhanced, and the T′ phase switches to negative under illumination	[145]
Morphotropic phase boundaries	BVO/YSZ	Enhanced photocurrent and photocatalytic performance at domain walls	[146]

*Note*: Macroscopic bending refers to strain gradient engineering methods that impose a global mechanical deformation on the entire sample.

#### 2.2.1 AFM‐Probe‐Induced Local Strain Gradients

**FIGURE 5:**
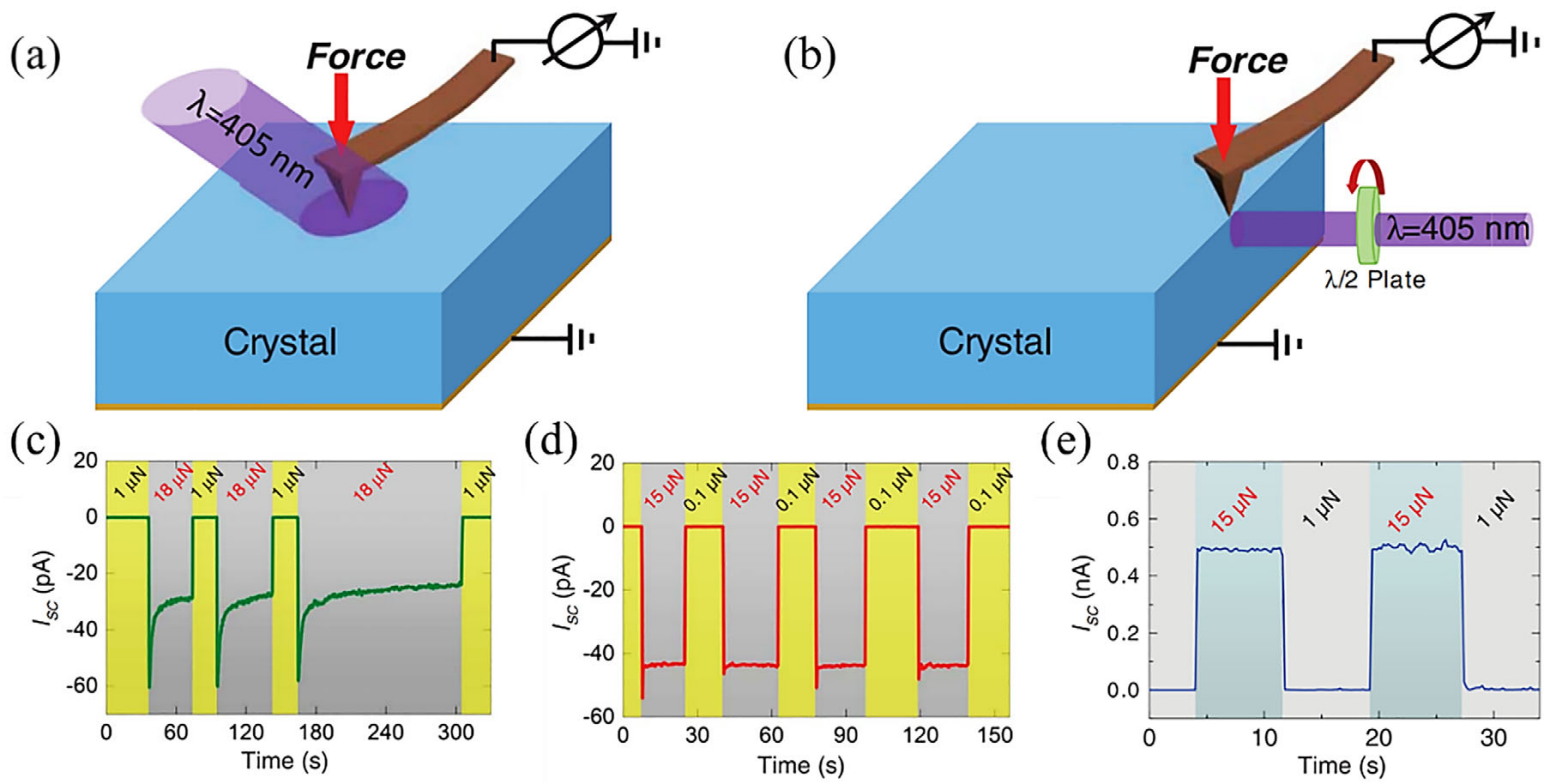
(a) Experiment setup for illumination around the contact area. (b) Experiment set‐up for illumination on the side surface to prevent Fresnel reflection and to ensure that light absorption would be independent of its polarization. Evidence of flexo‐photovoltaic effect in STO (c), TiO_2_ (d), and Si (e) under AFM probe pressing. Adapted with permission from ref. [94] Copyright 2018 The Authors, some rights reserved; exclusive licensee American Association for the Advancement of Science.

The term “flexo‐photovoltaic effect” was first coined by Yang et al. [94] to describe the phenomenon in which the short‐circuit current *I*_sc_ in centrosymmetric crystals increases by over two orders of magnitude under the localized pressure applied by an AFM probe. As illustrated in Figure 5, the experiment configurations were designed for contact‐area illumination (Figure 5a) and side‐surface illumination (Figure 5b), respectively, to avoid the influence of Fresnel reflection and light polarization. Under 405 nm laser illumination, the flexo‐photovoltaic response could be modulated by varying the applied force in the ranges of 1‐18 µN for STO (001) (Figure 5c), 0.1‐15 µN for TiO_2_ (100) (Figure 5d), and 1‐15 µN for Si (001) (Figure 5e). Considering that STO is a quantum paraelectric material capable of undergoing a cubic‐to‐tetragonal phase transition under high stress or strain gradient, it is plausible that perovskites exhibiting stress‐ or strain‐gradient‐induced phase transitions may possess larger effective flexoelectric coefficients, thereby enabling stronger flexo‐photovoltaic coupling. Despite its limitations, such as variable contact resistance due to changes in contact area and applied force, and reliance on the idealized assumption of spherical indenter‐induced strain (gradient) distribution, the AFM‐probe approach offers a novel perspective for studying mechanically enhanced photovoltaic effects in a wide range of dielectric materials, including centrosymmetric ones. However, the strain gradients generated by AFM indentation are inherently local and nonuniform, complicating the accurate extraction of flexoelectric coefficients. Moreover, this method remains challenging to scale for practical device applications.

#### 2.2.2 Macroscopic Bending of Thin Films or Devices

In the section focusing on bending‐induced flexo‐photovoltaic effects, a notable trend has emerged: many studies employ a freestanding film fabrication process. Interestingly, many thin‐film materials previously regarded as brittle can sustain remarkably large strain gradients when released from rigid substrates and transferred onto flexible supports. The freestanding approach unlocks the material's inherent potential by eliminating the clamping constraints imposed by the original substrate. Moreover, the transferability of freestanding films to diverse target substrates not only expands design flexibility but also offers a route more compatible with scalable manufacturing and practical device integration.

**FIGURE 6:**
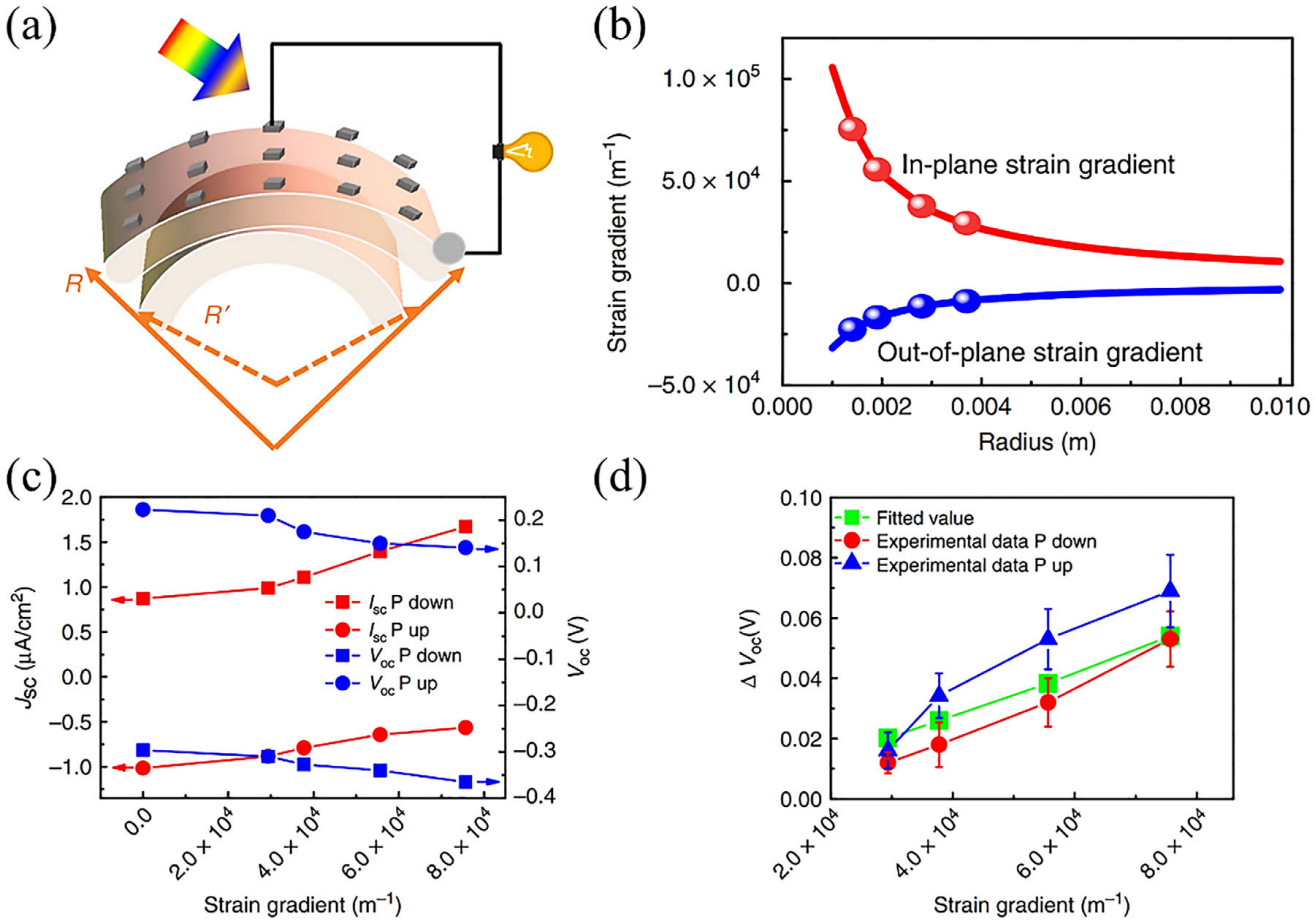
Continuous control of flexo‐photovoltaic effect in freestanding BFO via tunable bending: (a) Sketch of circuit under different bending radius; (b) Relationship between strain gradient and radius; (c) *J*_sc_ and (d) Δ*V*_oc_ under strain gradient ranging from 0 to 7.5 × 10^4^ m^−1^. Adapted with permission from ref. [95] Copyright 2020, CC BY 4.0.

The flexo‐photovoltaic effect becomes particularly promising in freestanding systems, where the removal of a rigid substrate affords enhanced mechanical flexibility and enables the sustainment of considerably larger strain gradients. Guo et al. [95] demonstrated continuously tunable photoconductance in freestanding BiFeO_3_ films. In their work, a BiFeO_3_ film epitaxially grown on a SrTiO_3_ substrate was transferred onto a flexible substrate by dissolving a sacrificial layer. Tunable flexoelectricity was achieved by bending the substrate, which induces nonuniform lattice distortion in the BiFeO_3_ layer and thereby modifies the inversion asymmetry of the film, as illustrated in Figure 6a,b. As a result, the short‐circuit current density *J*_sc_ (Figure 6c) and the variation of open‐circuit voltage *ΔV_oc_ (*Figure 6d) can be adjusted continuously through the strain gradient. This coupling between the flexoelectric and ferroelectric photovoltaic effects enables multilevel photoconductance states, which exhibit excellent reproducibility upon repeated bending cycles of the flexible BiFeO_3_ device. Adopting a flexible substrate for epitaxial growth is also a feasible strategy for a freestanding system. Jiang et al. [66] reported flexoelectric‐induced photovoltaic effects in centrosymmetric LaFeO_3_ thin‐film heterostructures grown on flexible mica substrates. Concave or convex bending of the mica substrate creates mechanically tunable strain gradients, enabling bidirectional modulation of the photocurrent. The short‐circuit current density exhibits reversible changes of approximately 100% in magnitude and shows excellent reproducibility over repeated bending cycles.

**FIGURE 7:**
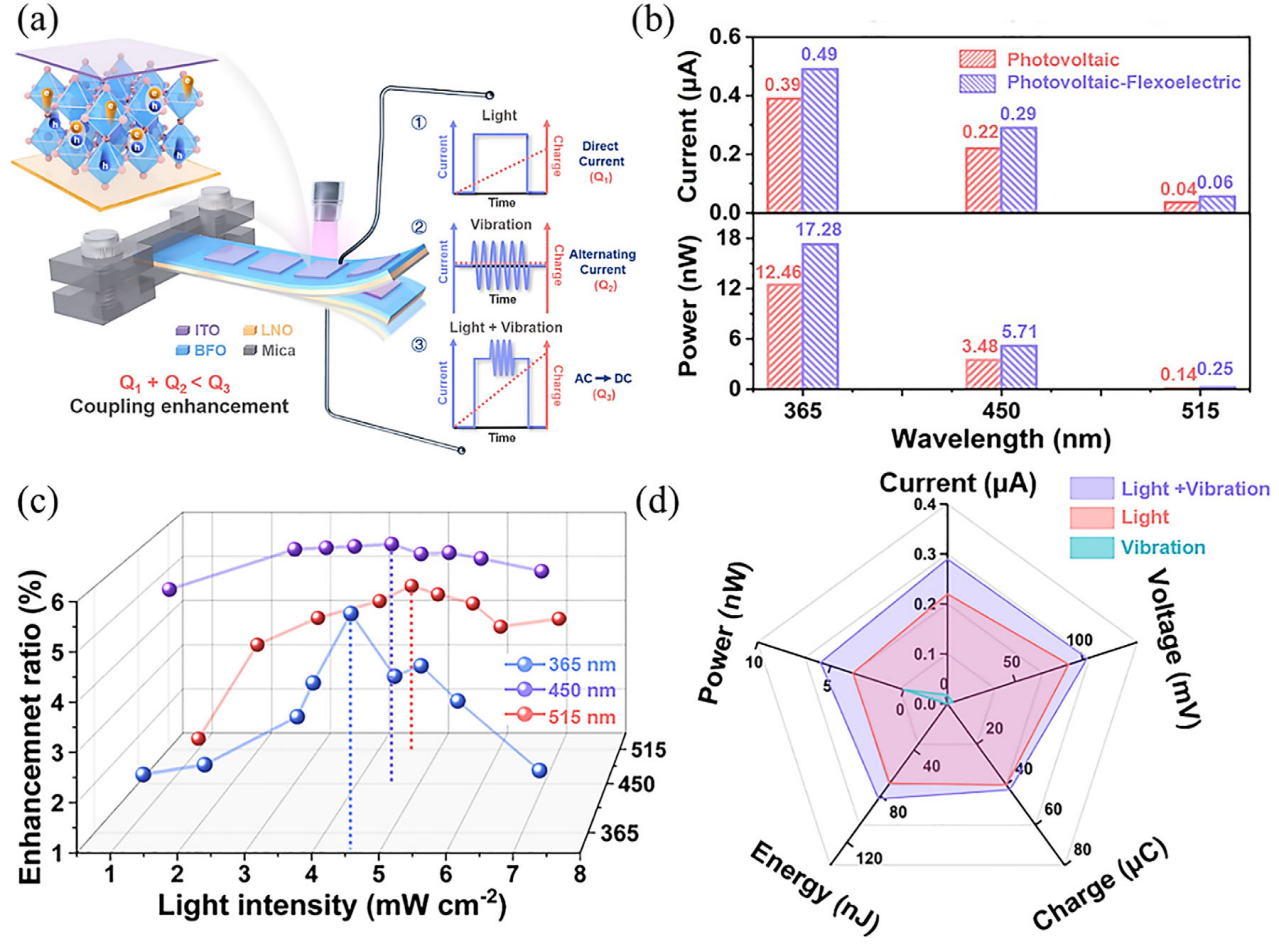
(a) Design of LNO/BFO/ITO nanogenerator. (b) Short‐circuit currents and output powers of devices caused by light and light‐vibration coupling. (c) Charge enhancement ratio as a function of light intensity. (d) Comprehensive performance comparison. Adapted with permission from ref. [90] Copyright 2022 The Author(s), under exclusive license to Springer Nature B.V.

Ferroelectric materials are well‐suited for multi‐energy harvesting applications owing to their intrinsic multi‐field coupling capabilities. By simultaneously harnessing mechanical vibration and light illumination, such systems can achieve significantly enhanced energy conversion efficiency through synergistic flexo‐photovoltaic and piezoelectric–pyroelectric coupling. Han et al. [90] developed an integrated nanogenerator based on a flexible BiFeO_3_ ferroelectric film that effectively couples flexoelectric and photovoltaic mechanisms, enabling the simultaneous harvesting of light and mechanical vibration energy, as illustrated in Figure 7a. Figure 7b shows that the device exhibits enhanced performance across a relatively broad range of the visible spectrum. Furthermore, as shown in Figure 7c, the device not only converts alternating current into direct current but also achieves a 6.2% enhancement in charge storage and a 19.3% improvement in energy output with the engagement of the flexoelectric effect. A more comprehensive analysis presented in Figure 7d reveals a multidimensional synergistic enhancement where the combined effect exceeds the sum of the individual contributions in terms of current, charge, and overall energy performance. Similarly, Wang et al. [134] reported enhanced power harvesting in a flexible potassium sodium niobate/poly (vinylidene fluoride‐trifluoroethylene) (KNN/PVDF‐TrFE) nanocomposite mediated by the flexo‐photovoltaic effect. When subjected to a curvature of 1/20, the *I*_p__v_ of the curved nanocomposite increased by about 13.9% compared to its flat counterpart. Under the same curvature, the *I*_p__v_ of the nanocomposite was 71.6% higher than that of a pristine PVDF‐TrFE film. Because the enhancement in photovoltaic output significantly exceeds the sole contribution from flexoelectricity‐induced polarization, the observed behavior was identified as the flexo‐photovoltaic effect. Likewise, Qian et al. [138] developed a temperature‐enhanced flexo‐photovoltaic coupled nanogenerator for the concurrent harvesting of both vibrational and light energy across a broad temperature range. This flexible device architecture exhibits synergistic enhancement under combined mechanical excitation and optical illumination. The observed enhancement is attributed to multiple synergistic factors, including thermal expansion, an elevated flexoelectric coefficient, reduced Schottky barrier height, bandgap narrowing, and increased charge carrier mobility. Zhang et al. [135] systematically investigated the flexoelectric‐enhanced photovoltaic effect in curved 3D‐printed BaTiO_3_/PVDF composite films. Except for achieving a 3.7‐fold increase over pristine PVDF under bending and illumination, when implemented as flexible ferroelectric memories, the BTO/PVDF‐based devices exhibited a signal intensity difference between logic “0” and “1” states exceeding tenfold, enabling direct readout using a laser.

**FIGURE 8:**
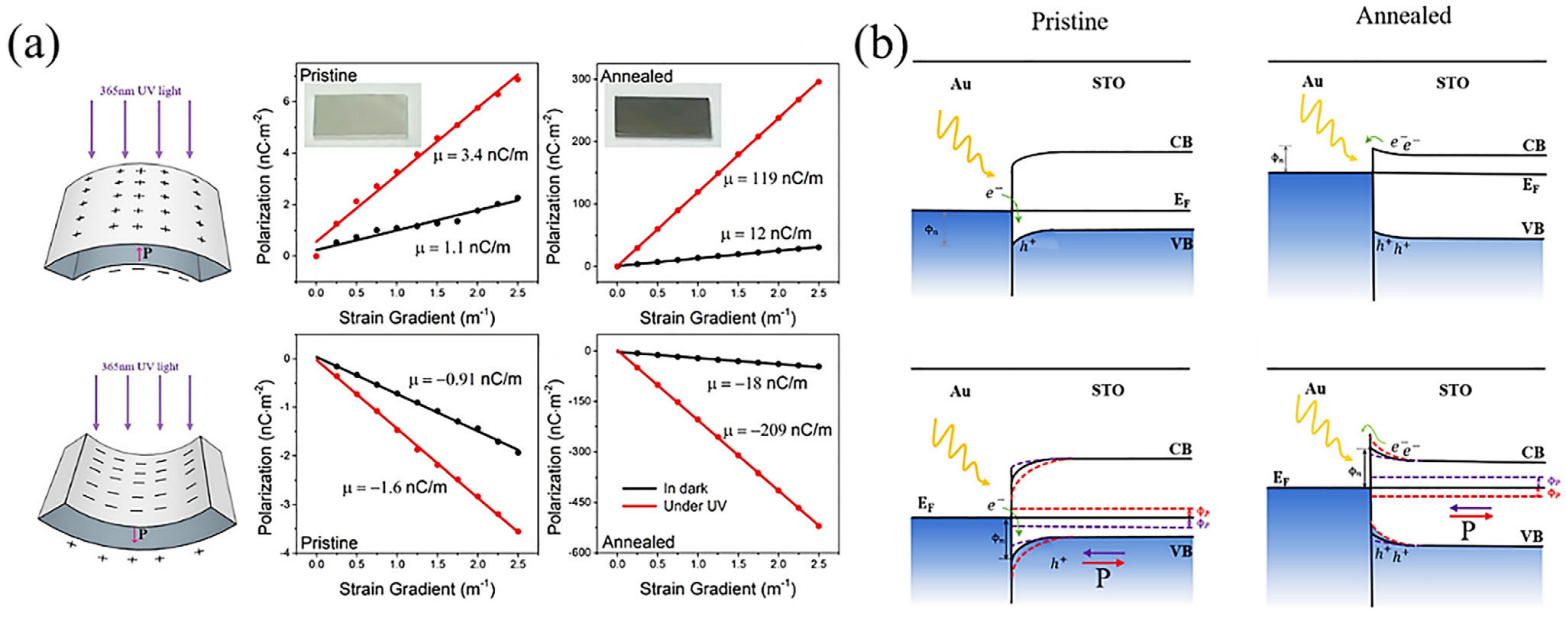
(a) Enhanced effective flexoelectric effect under UV light of pristine and annealed STO sample under n and u‐shape bending. (b) Energy band diagrams and electron tunneling at the Au/SrTiO_3_ interface for pristine and annealed STO samples. Adapted with permission from ref. [76] Copyright 2021 AIP Publishing LLC.

Single crystals provide an ideal platform for investigating fundamental coupling mechanisms, such as the flexo‐photovoltaic effect, due to their high purity and well‐defined crystallographic orientations. While they may not withstand large externally applied strain gradients, their structural uniformity and minimal defect density make them invaluable for elucidating the intrinsic physical processes underlying strain‐gradient‐driven photovoltaic phenomena. Jin et al. [76] demonstrated the photo‐flexoelectric effect in perovskite STO single crystals and elucidated the coupling mechanism between its photovoltaic and flexoelectric behaviors. As shown in Figure 8a, under illumination, light‐induced electrons tunnel through the Au/STO Schottky junction driven by the flexoelectric field, leading to a significant enhancement in flexoelectric response and the photo‐flexoelectric effect. Vacuum annealing introduced oxygen vacancies into STO, which strengthened light absorption and further amplified the photo‐flexoelectric output. The enhancement was achieved by bandgap engineering through oxygen vacancy doping, as shown in Figure 8b. The study revealed that by combining UV illumination and oxygen vacancy engineering, the effective flexoelectric coefficient of STO could be enhanced by over two orders of magnitude compared to that of stoichiometric crystals.

**FIGURE 9:**
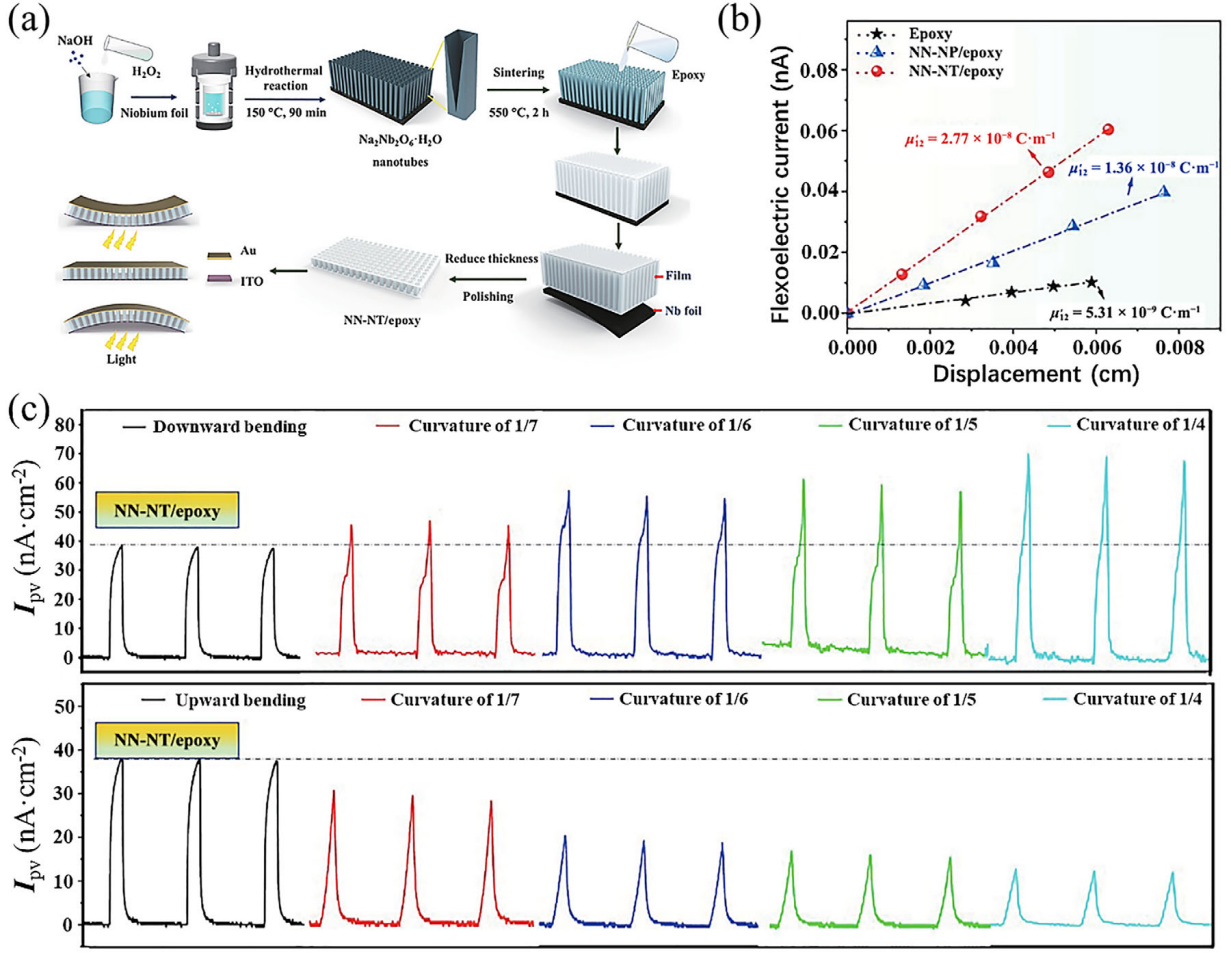
(a) Preparation of NN‐NT nanotubes by the hydrothermal method. (b) Linear relationship between the flexoelectric current and tip displacement. (c) *I*_pv_ of the NN‐NT/epoxy at downward and upward bending. Adapted with permission from ref. [137] Copyright 2023 The Author(s), under exclusive license to Tsinghua University Press and Springer‐Verlag GmbH.

Nanotubes and nanowires are widely recognized as promising materials for flexible applications, including vibration energy harvesting. The integration of flexo‐photovoltaic coupling in such nanostructures has further enhanced their energy‐harvesting efficiency by enabling simultaneous conversion of mechanical strain gradients and light into electrical energy. Yu et al. [137] synthesized vertically aligned NaNbO_3_ nanotubes (NN‐NT) with a trapezoidal morphology, exhibiting coexistence of orthorhombic and monoclinic phases. The fabrication process is illustrated in Figure 9a. Structural analysis indicated that the NN‐NT/epoxy composite film possesses remarkable flexoelectric properties, attributed to lattice distortions induced by structural defects and non‐uniform nanotube geometry. The flexoelectric response was most pronounced along the vertical direction, with a measured flexoelectric coefficient (*µ*_12_) of 2.77 × 10^−8^ C/m, approximately five times greater than that of pure epoxy, as shown in Figure 9b. As illustrated in Figure 9c, under downward bending, the photovoltaic current *I*_pv_ of the composite film increased from 39.9 to 71.8 nA/cm^2^ along the spontaneous polarization direction, clearly demonstrating a flexoelectricity‐enhanced photovoltaic effect. According to the real‐time current data, a slight decrease showed up before *I*_pv_ reached the peak, which might arise from the released surface charges during the measurement or screening charges response to the downward polarization induced by the flexoelectric effect. Moreover, mechanical bending enabled reversible modulation of the photovoltaic response, enhancing it by up to 80% or suppressing it to 65% of its initial value. Similarly, Zhang et al. [136] reported a flexoelectricity‐enhanced photovoltaic effect in a flexible nanocomposite consisting of a Pb(Zr_0.52_Ti_0.48_)O_3_ nanowire (PZT NW) array embedded in a polydimethylsiloxane (PDMS) matrix. When the nanocomposite film was bent downward, the photovoltaic current of the aligned PZT‐NW/PDMS composite increased by 84.6 times and 27.6 times compared to PZT‐nanoparticle/PDMS and randomly aligned PZT‐NW/PDMS composites under the same curvature, respectively. This enhancement is primarily attributed to the increased flexoelectric response in the aligned nanowire composite.

#### 2.2.3 Spontaneously Formed Strain Gradients

Epitaxially grown thin films can develop strain gradients along the vertical‐thickness direction through lattice‐mismatch strain relaxation. This intrinsic strain gradient inherently introduces a flexoelectric field, which enhances carrier separation without requiring external mechanical actuation. Furthermore, the epitaxial growth process generally ensures high crystalline quality and clean interfaces, offering additional advantages in terms of structural coherence and optoelectronic performance.

**FIGURE 10:**
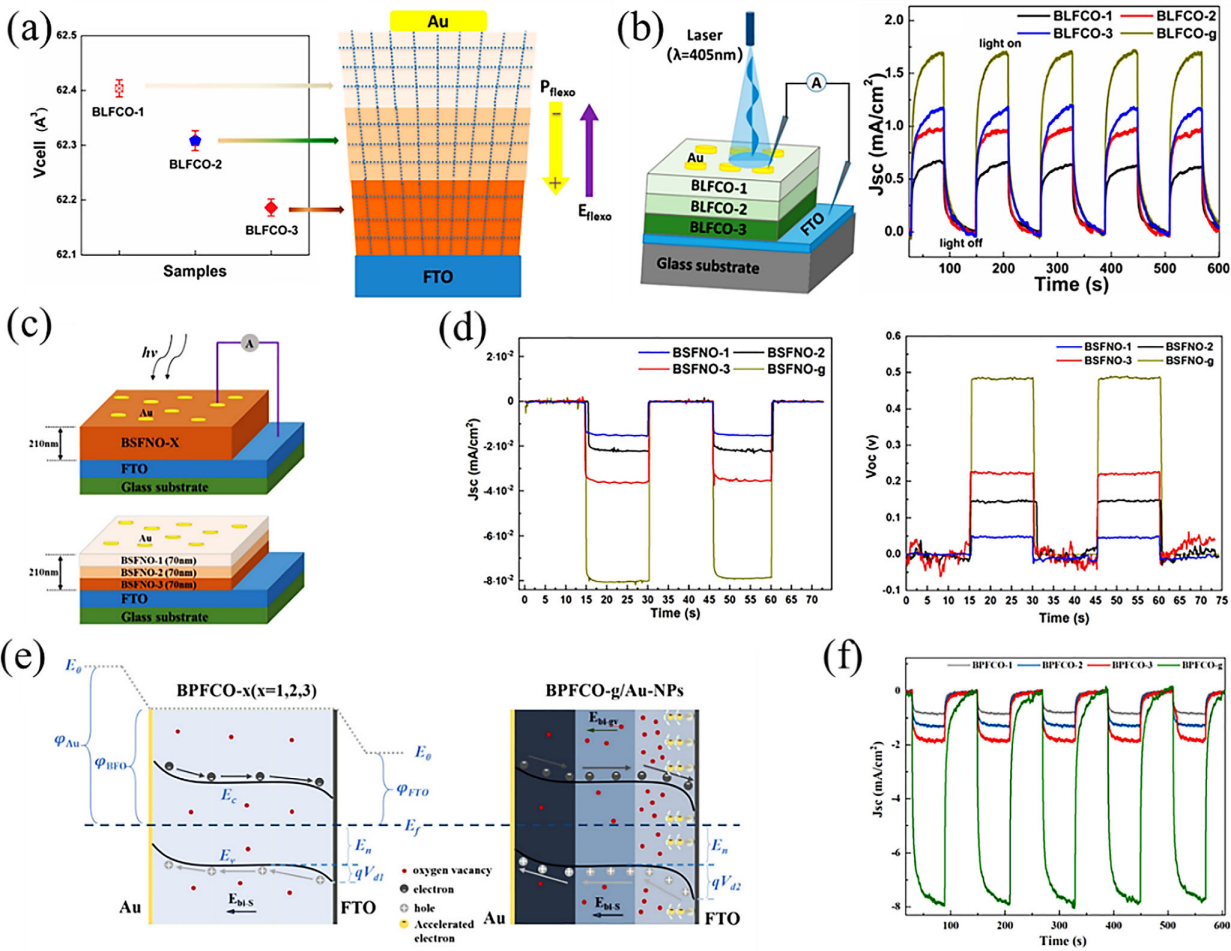
(a) Cell volume of BLFCO‐1, ‐2, and ‐3. (b) Schematic diagram for photovoltaic measurements and time‐dependent photocurrent density curves for BLFCO‐*x* (*x* = 1, 2, 3, and *g*) with light on and off. (c) Schematic diagram of BSFNO photovoltaic device and (d) *J*_sc_, *V*_oc_ test of BSFNO thin films. (e) Energy band diagrams of conventional doped samples without the LSPR effect and gradient doped samples with the LSPR effect. (f) *J*_sc_‐*T* curves of BPFCO*‐x* (*x *= 1, 2, 3, and *g*)/Au‐NPs heterostructures under alternating illumination. (a,b) Adapted with permission from ref. [139] Copyright 2021 American Chemical Society. (c,d) Adapted with permission from ref. [140] Copyright 2023 the Partner Organizations. (e,f) Adapted with permission from ref. [141] Copyright 2024 the Partner Organizations.

The growing recognition of the anomalous ferroelectric photovoltaic effect in multiferroic BFO, often attributed to flexo‐photovoltaic mechanisms, has motivated research to address the critical issue of low photocurrent density, alongside efforts in bandgap engineering for specific spectral ranges and junction designs. In this context, Sun et al. [139] developed a novel strategy to enhance the photocurrent density of BiFeO_3_ by leveraging the cooperative effects of a gradient distribution of oxygen vacancies and the resulting flexoelectric effect. This was achieved through (La, Co) gradient‐doped BiFeO_3_ (BLFCO) multilayers, as shown in Figure 10a. Subsequent measurements and analysis revealed that the gradient‐doped multilayer BiFeO_3_ exhibited a photocurrent density nearly three times higher than that of a conventionally doped single‐layer counterpart, as demonstrated in Figure 10b. Using a related approach, Sun et al. [141] further achieved enhanced photovoltaic performance in (Sm, Ni) gradient‐doped BiFeO_3_ (BSFNO) multilayers with an oxygen vacancy gradient, as shown in Figure 10c. The authors designed and fabricated a novel photovoltaic architecture, in which the graded distribution of oxygen vacancies could significantly improve the photovoltaic response. As presented in Figure 10d, the gradient‐doped multilayer exhibited a short‐circuit current density *J*_sc_ of 80 µA/cm^2^ and an open‐circuit voltage *V*_oc_ of 0.49 V, substantially exceeding the values previously reported for pure BiFeO_3_ thin films. In a subsequent study, Sun et al. [141] employed a multi‐strategy approach to further enhance the photovoltaic performance of BiFeO_3_‐based devices. They constructed an elaborate device with an Au/BPFCO‐g/Au‐NPs/FTO architecture, incorporating a (Pr, Co) gradient‐doped BiFeO_3_ (BPFCO‐g) multilayer film with an Au nanoparticle layer to synergistically enhance charge separation and collection. The energy band diagram is shown in Figure 10e. The resulting photovoltaic device exhibited a dramatically enhanced photocurrent density, reaching 8 mA/cm^2^, which is approximately 727 times higher than that of a pure BiFeO_3_ film (∼0.011 mA/cm^2^), as shown in Figure 10f. Notably, the photocurrent showed negligible degradation during a long‐term stability test lasting 1800 s, demonstrating the exceptional durability and repeatability. Huang et al. [142] demonstrated significant modulation of the ferroelectric photovoltaic effect via a giant macroscopic flexoelectric effect induced by strain‐relaxed epitaxy. By employing this approach, they generated and tuned giant strain gradients exceeding 10^7^ m^−1^ in ferroelectric Pb(Zr_0.2_Ti_0.8_)O_3_ epitaxial thin films. Tuning these strain gradients enabled substantial modification of the switchable ferroelectric photovoltaic properties, resulting in a photovoltage enhancement of approximately 0.5 V. The authors proposed that this flexoelectric modulation of photovoltaic behavior might originate from a flexoelectric polarization‐induced depolarization field. Vayalil et al. [143] also explored bandgap engineering via the flexo‐photovoltaic effect. In their work, a strain gradient‐induced flexoelectric field was utilized to enhance and sustain the photovoltaic response at elevated temperatures. This mechanism, termed the flexo‐photovoltaic effect, was demonstrated in a bandgap‐tuned 0.95BaTiO_3_‐0.05Bi(Ni_0.5_Nb_0.5_)O_3+δ_ film grown epitaxially under compressive strain on a SrRuO_3_‐buffered SrTiO_3_(001) substrate.

**FIGURE 11:**
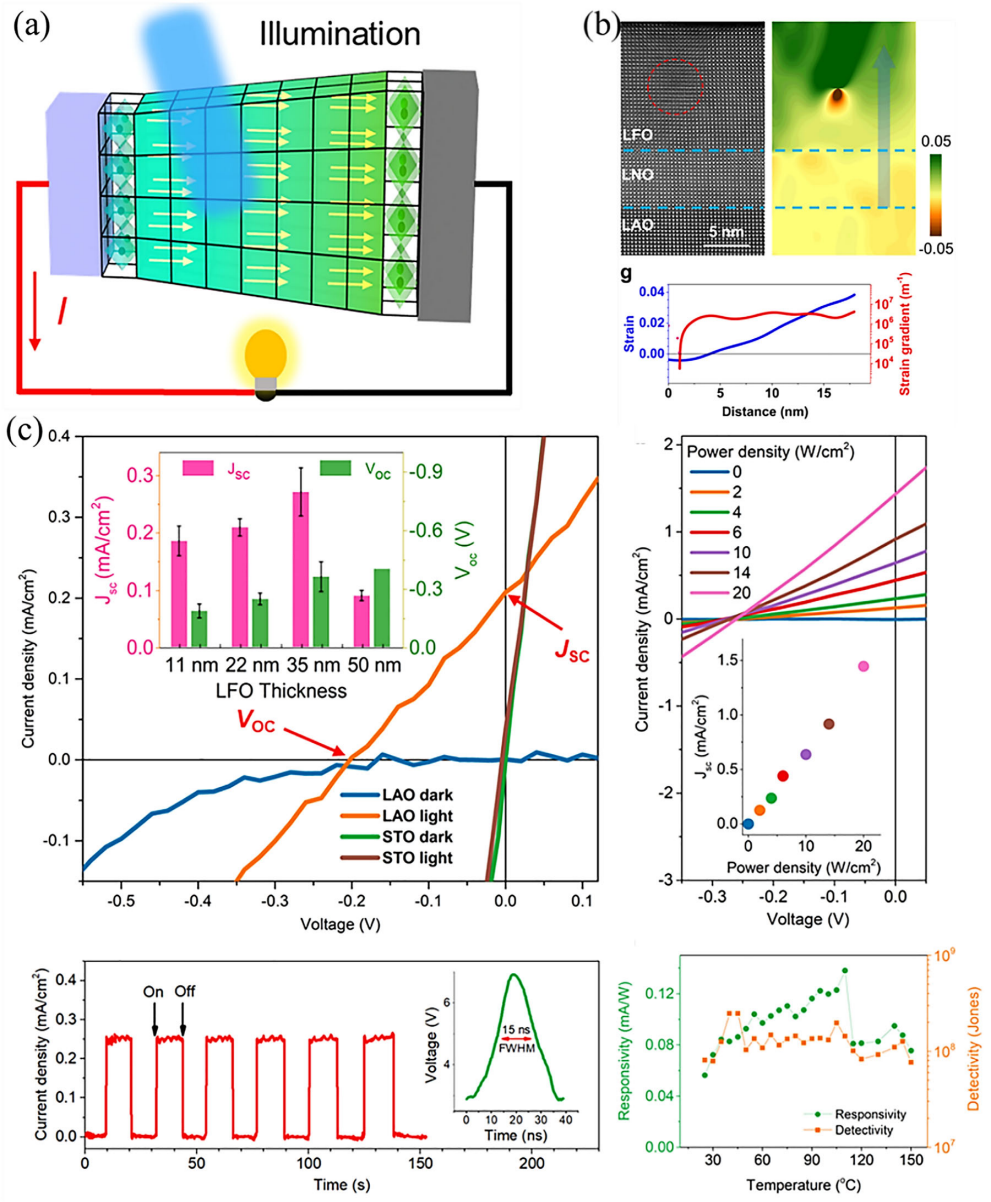
(a) The relaxation of mismatch strain in the epitaxial structure. (b) TEM image, strain, and its gradient of LFO/LNO/Lao. (c) Comprehensive photo‐response of Pt/LFO/LNO/LAO. Adapted with permission from ref. [97] Copyright. 2021 American Chemical Society.

The flexoelectric effect can generate a significant internal electric field, functionally analogous to that of a conventional *p–n* junction. Wu et al. [97] proposed a flexoelectric photodetector based on a thin‐film heterostructure, demonstrated using epitaxial LaFeO_3_ films grown on LaAlO_3_ substrates, as shown in Figure 11a. A giant strain gradient on the order of 10^6^ m^−1^, as illustrated in Figure 11b, was achieved in the LaFeO_3_ layer, inducing strong flexoelectric polarization and resulting in a pronounced photovoltaic effect in the heterostructure with a nanosecond‐scale photoresponse under illumination. As shown in Figure 11c, the resulting Pt/LFO/LNO/LAO device exhibited a spontaneous photo‐response with a responsivity of 10^−4^ A/W, a detectivity of 10^8^ Jones, and a response time of 15 ns.

**FIGURE 12:**
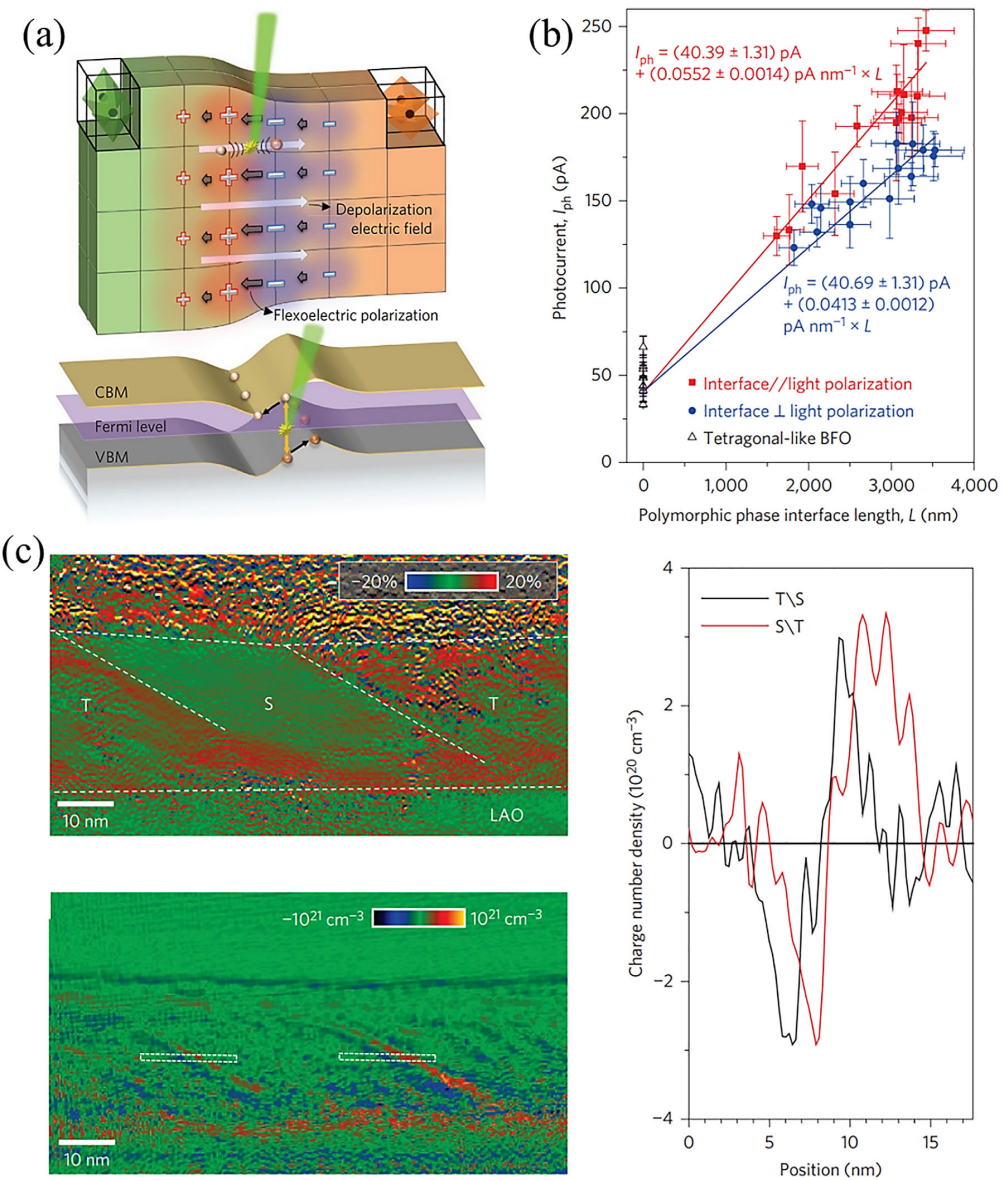
(a) Schematic of the strain gradient at the interface and the effect of the flexoelectric effect on the band diagram. (b) The average photocurrent of interfaces parallel and vertical to light polarization. (c) Experimental data of strain and total charge density maps of a mixed‐phase area obtained from the phase information of the reconstructed exit wavefunction of the transmitted beam. Adapted with permission from ref. [93] Copyright. 2015 Springer Nature Limited.

Multiferroic materials serve as a versatile platform for multi‐field coupling, as their spontaneous order parameters, including electric polarization and magnetic moments, are inherently coupled to the crystal lattice. This intrinsic coupling enables rich interactions among light, heat, mechanical force, and electromagnetic fields. Consequently, domain patterns, including domain walls and phase boundaries, often host a variety of emergent physical phenomena, making them fertile ground for exploring novel opto‐mechano‐electric effects. Chu et al. [93] observed enhanced photocurrent at domain walls in mixed‐phase BiFeO_3_ films grown on LaAlO_3_ (LAO) substrates, as shown in Figure 12a. The lattice mismatch strain induced a morphotropic phase boundary (MPB) between tetragonal‐like (T‐BFO) and rhombohedral‐like (S‐BFO) phases, which differ structurally, thereby generating a pronounced strain gradient as well as a flexoelectric effect across their charged interface, as illustrated in Figure 12c. As shown in Figure 12b, this dipole at the domain walls, confirmed through both experimental and theoretical studies, creates a strong built‐in electric field that effectively separates photoexcited electron–hole pairs, leading to the observed photocurrent enhancement.

Similarly, a subsequent investigation by Yang et al. [145] on the BFO/LAO system further underscored the critical influence of strain gradients on governing local photoelectric properties. Using nanoscale‐resolution measurements of local conduction under both dark and illuminated conditions, the study revealed that the substantial strain gradient at the MPBs activates a pronounced flexo‐photovoltaic effect. This effect simultaneously enhances photoconduction in the rhombohedral R‐phase while inducing negative photoconductivity in the morphotropic T′‐phase. While charged domain walls have been extensively studied in BiFeO_3_, a related phenomenon was reported by Shao et al. [146] in centrosymmetric epitaxial BiVO_4_ films, where they identified ferroelastic twin textures with localized flexoelectric effects. Through photodeposition experiments and localized photocurrent measurements, the authors demonstrated that the flexoelectric fields confined to domain walls facilitate photo‐induced charge carrier transport. This flexo‐phototronic mechanism was further corroborated by dye‐degradation tests and the detection of reactive radicals.

The flexo‐photovoltaic effect can also be achieved in ceramic systems. Gong et al. [144] investigated the photovoltaic properties of both ferroelectric (Bi_0.5_Na_0.5_)TiO_3_ (BNT) and paraelectric 0.3(Bi_0.5_Na_0.5_)TiO_3_‐0.7BiFeO_3_ (0.3BNT‐0.7BFO) ceramics, attributing the anomalous photovoltaic (APV) effect observed in the paraelectric composition to flexo‐photovoltaics. Their results indicated that pure BNT, owing to its intrinsic ferroelectricity, exhibits a pronounced photovoltaic response even under 550 nm visible‐light illumination. In contrast, the 0.3BNT‐0.7BFO solid solution, despite its enhanced visible‐light absorption, shows no ferroelectric photovoltaic contribution due to its negligible ferroelectricity; instead, it demonstrates a strong strain‐induced flexo‐photovoltaic effect.

### 2.3 Section Summary

Research on flexo‐photovoltaic effects in oxide perovskites can be systematically categorized according to the origin of the strain gradient. Studies utilizing AFM‐probe‐induced local strain have provided foundational insights into strain‐gradient‐driven photovoltage enhancement, even in centrosymmetric systems; however, their scalability remains limited. In contrast, works based on macroscopic bending, which often employ freestanding films transferred onto flexible substrates, have demonstrated that large, tunable strain gradients can significantly enhance photovoltaic and photoconductive responses, highlighting a more practical and scalable route for strain engineering. Meanwhile, investigations of intrinsic strain gradients arising from lattice‐mismatch relaxation, composition grading, grain boundaries, or ferroelastic domain walls reveal that built‐in flexoelectric fields can persistently promote charge‐carrier separation, offering a materials design strategy for intrinsically enhanced optoelectronic performance. Collectively, these case studies illustrate that strain gradients, whether externally applied or intrinsically generated, can effectively break symmetry and enhance photoconversion in oxide perovskites, with freestanding architectures emerging as a particularly promising platform for functional device integration.

## 3 Flexo‐Photoconversion in Halide Perovskites

### 3.1 Material Systems and Their Features

Research reports on halide perovskites in modern photovoltaics did not originate from a deliberate search for a revolutionary solar absorber, but rather from their initial use as novel, solution‐processable semiconductors in dye‐sensitized solar cells. The pivotal breakthrough came in 2009, when Miyasaka and colleagues first employed lead–iodide‐based hybrid perovskite (CH_3_NH_3_PbI_3_ and CH_3_NH_3_PbBr_3_) as light‐sensitive dyes, achieving a modest efficiency of around 3.8% [147]. However, these liquid‐electrolyte‐based devices suffered from severe instability. The field truly ignited in 2012 with the seminal work of replacing the liquid electrolyte with a solid‐state hole transporter, which dramatically boosted both efficiency and stability, pushing efficiencies above 9% and later to 10% [148, 149]. This advance unleashed a torrent of global research, leading to an unprecedented meteoric rise in certified PCEs, which now exceed 26% for single‐junction cells [150], rivaling established silicon technology.

**FIGURE 13:**
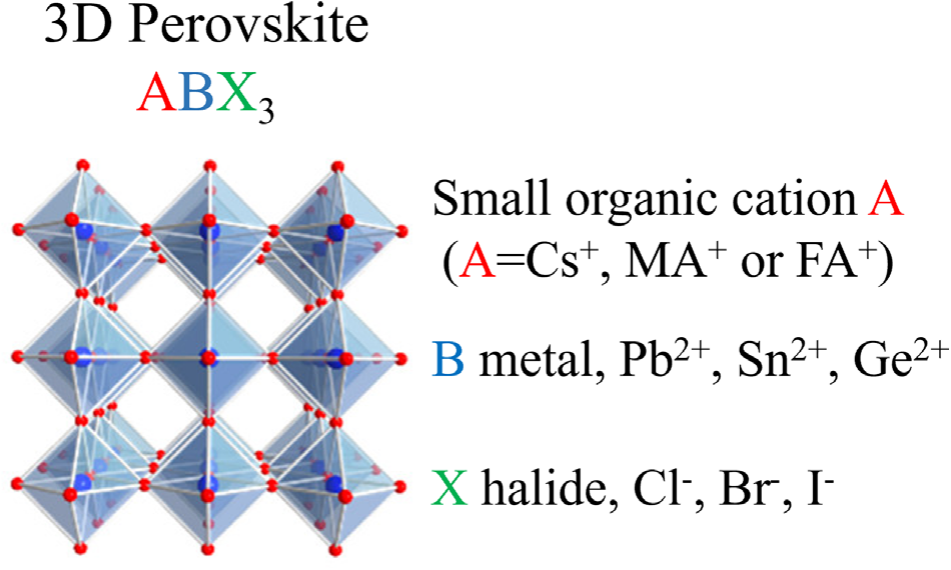
Structure of typical halide perovskites. Adapted with permission from ref. [151] Copyright. 2018 American Chemical Society.

While having a diverse chemical composition, halide perovskites are commonly represented by the general formula *ABX*_3_, where ‘*A*’ is an organic cation like methylammonium (M*A* ^+^) or formamidinium (F*A* ^+^) or metal cation like Cs^+^, ‘*B*’ is a metal cation (typically Pb^2+^ or Sn^2+^), and ‘*X*’ is a halide anion (I^−^, Br^−^, Cl^−^) [151], as shown in Figure 13. Structurally, these materials share a defining corner‐sharing BX_6_ octahedral framework that forms a periodic lattice, like that of traditional oxide perovskites, with the ‘*A*’ cations residing in the cuboctahedra cavities of the inorganic framework. However, due to the structural flexibility introduced by organic cations, the origin of lattice‐related properties, such as ferroelectricity, in hybrid organic–inorganic halide perovskites differs significantly from that in their all‐inorganic counterparts. In these hybrid systems, ferroelectric behavior may arise not only from ion displacement within the inorganic framework but also from order–disorder transitions of organic moieties and even from ion migration dynamics, reflecting a more complex and multifaceted interplay between organic and inorganic sublattices [152]. This unique structure gives rise to a suite of extraordinary physical properties, including exceptionally high optical absorption coefficients, allowing for very thin, lightweight active layers; long charge‐carrier diffusion lengths combined with low exciton binding energies, enabling efficient charge carrier transport and collection [153, 154, 155]; widely tunable bandgaps (from ∼1.5 to over 3.2 eV) via simple halide or cation mixing [156, 157, 158]; and remarkable defect tolerance, where high‐performance devices can be made even with relatively low‐cost, solution‐based processing. Beyond photovoltaics, these properties have made halide perovskites a compelling material platform for diverse optoelectronic applications, including bright and color‐tunable light‐emitting diodes, lasers, and photodetectors [159, 160, 161, 162, 163, 164, 165].

The investigation of the flexoelectric effect in halide perovskites is a relatively nascent but rapidly evolving research field, emerging as a corollary to the intense research on their photovoltaic and optoelectronic properties. Initially, the remarkable electronic and optical characteristics of materials like MAPbI_3_ were primarily attributed to their defect tolerance and optimal band structures [166]. However, by the mid‐2010s, as the understanding of these soft, ionic semiconductors deepened, researchers began to systematically explore their electromechanical properties [167]. The recognition that halide perovskites possess exceptionally low elastic moduli and high dielectric constants is the key parameters that theoretically predict a giant flexoelectric response, sparking focused investigations. Early theoretical studies and nanoscale measurements, often using conductive atomic force microscopy (c‐AFM) to induce localized strain gradients, confirmed that these materials could indeed generate substantial flexoelectric polarization [98, 168]. These findings established that the flexoelectric effect is not merely a secondary phenomenon in these systems, but rather a significant contributor to the complex electronic and functional landscape of halide perovskites.

The evolving research further indicates that the effective flexoelectric coefficients in halide perovskites, such as MAPbI_3_ and MAPbBr_3_, significantly exceed those of conventional oxide perovskites like SrTiO_3_, particularly under illumination [78]. The precise intrinsic bulk flexoelectric coefficients of halide perovskites remain undetermined; however, as experimentally reported values represent effective coefficients that incorporate both bulk and surface contributions, with surface effects often dominant in semiconducting systems. Studies suggest that ferroelectric and flexoelectric responses in these materials are strongly influenced by cation rotation and even migration, making it inherently challenging to isolate and quantify the true flexoelectric coefficient for a given halide perovskite.

Halide perovskites have revolutionized the field of photovoltaics due to their exceptional optoelectronic properties, including strong light absorption, long carrier diffusion lengths, and highly tunable bandgaps. For single‐junction solar cells, the bandgap of these perovskites is typically engineered to be in the range of approximately 1.5‐1.6 eV, which is near‐ideal for solar energy harvesting. Devices with such bandgaps can achieve impressive *V*_oc_ often between 1 and 1.28 V, with short‐circuit current densities *J*_sc_ frequently exceeding 25 mA/cm^2^. These have contributed to certified PCEs for single‐junction lab‐scale cells soaring from 3.8% to over 26% within just over a decade [147, 150]. Currently, the most suitable absorber materials are multi‐cation compositions based on formamidinium lead iodide (FAPbI_3_), often stabilized with cesium (Cs) and methylammonium (MA), as they offer a favorable balance among bandgap, stability, and device performance.

To push beyond the efficiency limits of single‐junction cells, halide perovskites have been brilliantly deployed in multi‐junction (tandem) solar cell architectures. In such designs, a wide‐bandgap perovskite top cell (typically around 1.67 eV) is combined with a narrow‐bandgap bottom cell, such as silicon or a low‐bandgap perovskite. The wide‐bandgap perovskite is crucial for achieving a high *V*_oc_ (e.g., 1.28 V at a 1.67 eV bandgap) while allowing lower‐energy photons to reach the bottom sub‐cell. This tandem strategy has proven immensely successful, with certified efficiencies for monolithic perovskite/silicon tandem cells now reaching a remarkable 30.5% [169]. In parallel, all‐perovskite tandem cells, which use different perovskite compositions for the top and bottom sub‐cells, are also a rapidly advancing and important strategy for surpassing the efficiency limits of single‐junction devices.

While the exceptional photovoltaic performance of halide perovskites is well‐established, a complete understanding of their charge separation dynamics must extend beyond traditional semiconductor models. The unique soft and ionic lattice of these materials, which facilitates their remarkable optoelectronic properties, also renders them highly susceptible to strain gradients. This introduces a crucial, yet sometimes overlooked, mechanism: the coupling between flexoelectricity and photoconversion. The giant flexoelectric effect inherent to these soft perovskites can generate substantial polarization under strain, creating a powerful internal field that actively aids in the separation of photogenerated electron‐hole pairs. Consequently, exploring this flexo‐photovoltaic coupling is essential for unraveling the full picture of their high performance and for devising new strategies to further enhance device efficiency through deliberate strain‐gradient engineering.

### 3.2 Progress, Strategies, and Mechanisms

Following the classification framework applied in the previous case studies, the relevant literature in this section is organized according to the origin of the strain gradient. The related categories are: (a) macroscopic bending of thin films or devices, such as cantilever or three‐point bending; (b) AFM‐tip‐induced local strain gradient; and (c) spontaneously formed strain gradients at material boundaries or interfaces. The magnitude, repeatability, scalability, strengths, and drawbacks of strain gradient induction methods are listed in Tables 4 and 5, respectively.

**TABLE 4:** Magnitude, repeatability, and scalability of various strain gradient induction methods in halide perovskite materials.

Source of strain gradient	Magnitude (m^−1^)	Repeatability	Scalability
AFM probe pressing	10^7^	Low	Very low
Cantilever bending	10^−2^–10^1^	High	Moderate
Phase transition		Low–moderate	High
Grain boundaries	10^9^	Low	High

**TABLE 5:** Strengths and drawbacks of various strain gradient induction methods in halide perovskite materials.

Source of strain gradient	Strengths	Drawbacks
AFM probe pressing	Large localized strain gradient in situ local measurement	Downward strain gradient only only applied to the surface area
Cantilever bending	Suitable for dynamic test simple simulation model	Small strain gradient flexible samples required
Phase transition	Multi‐field control available high contrast between two states	Limited working temperature hysteresis and fatigue
Grain boundaries	Large localized strain gradient no external loading required	Poor reproducibility and uniformity potential defects

For halide perovskites, the introduction of strain gradients faces distinct challenges due to the soft and ionically active nature of these materials. Macroscopic cantilever bending offers high repeatability and well‐defined global strain gradients, making it suitable for quantitative flexoelectric studies, although its scalability remains moderate. AFM tip loading suffers from poor repeatability and scalability, as the soft surface is easily damaged and local indentation leads to irreversible deformation. Phase‐transition‐induced strain provides high scalability but shows only moderate repeatability due to thermal cycling fatigue and material degradation. Grain boundaries naturally exist in polycrystalline films and offer excellent scalability, yet their stochastic distribution results in low repeatability. Overall, cantilever bending is preferred for controlled fundamental studies, while grain boundaries and phase transitions are more relevant for scalable device applications despite their lower repeatability. Detailed strengths and drawbacks are shown in Table 5, and a detailed summary of studies on these topics is summarized in Table 6.

**TABLE 6:** Summary of flexo‐photoconversion studies in halide perovskites.

Source and magnitude of strain gradient	Material system	Properties/phenomenon	Ref
Single‐clamped cantilever bending ∼10^−2^ m^−1^	MAPbCl_3_, MAPbBr_3_ single crystal	Flexoelectric coefficients enhanced by over 10^2^ under illumination	[78]
Single‐clamped cantilever bending ∼10^−1^ m^−1^	MAPbBr_3_, MAPbI_3_ single crystal	Adjustable *V*_oc_ and *I*_sc_ from −0.06 to −0.14 V and 2.25 to 3.75 mA/m^2^ through bending, respectively. *V*_oc_ beyond the bandgap achievable	[98]
Composition gradient and phase transition	CsPb(Br_1‐x_Cl_x_)_3_ Nanowires	Enhanced exciton lifetime	[170]
In situ uniform curvature bending ∼10^5^ m^−1^	MAPbI_3_ nanowire	Hydrogen generation enhanced by more than 300% comparing to pure mechanocatalysis or photocatalysis	[171]
AFM probe pressing ∼10^7^ m^−1^ Uniform bending ∼20 m^−1^	NiO/MAPbBr_3_/PCBM	Solar cell efficiency regulation from 2% to 900% using an AFM probe comparing to the initial contact of force 0.5 µN, and −20% to +20% compared to the flat state by bending.	[172]
Grain boundaries ∼10^9^ m^−1^	CsPbX_3_	Enhanced photovoltaic shift current of ∼15 µA/V^2^ at grain boundaries	[92]

#### 3.2.1 Macroscopic Bending of Thin Films or Devices

**FIGURE 14:**
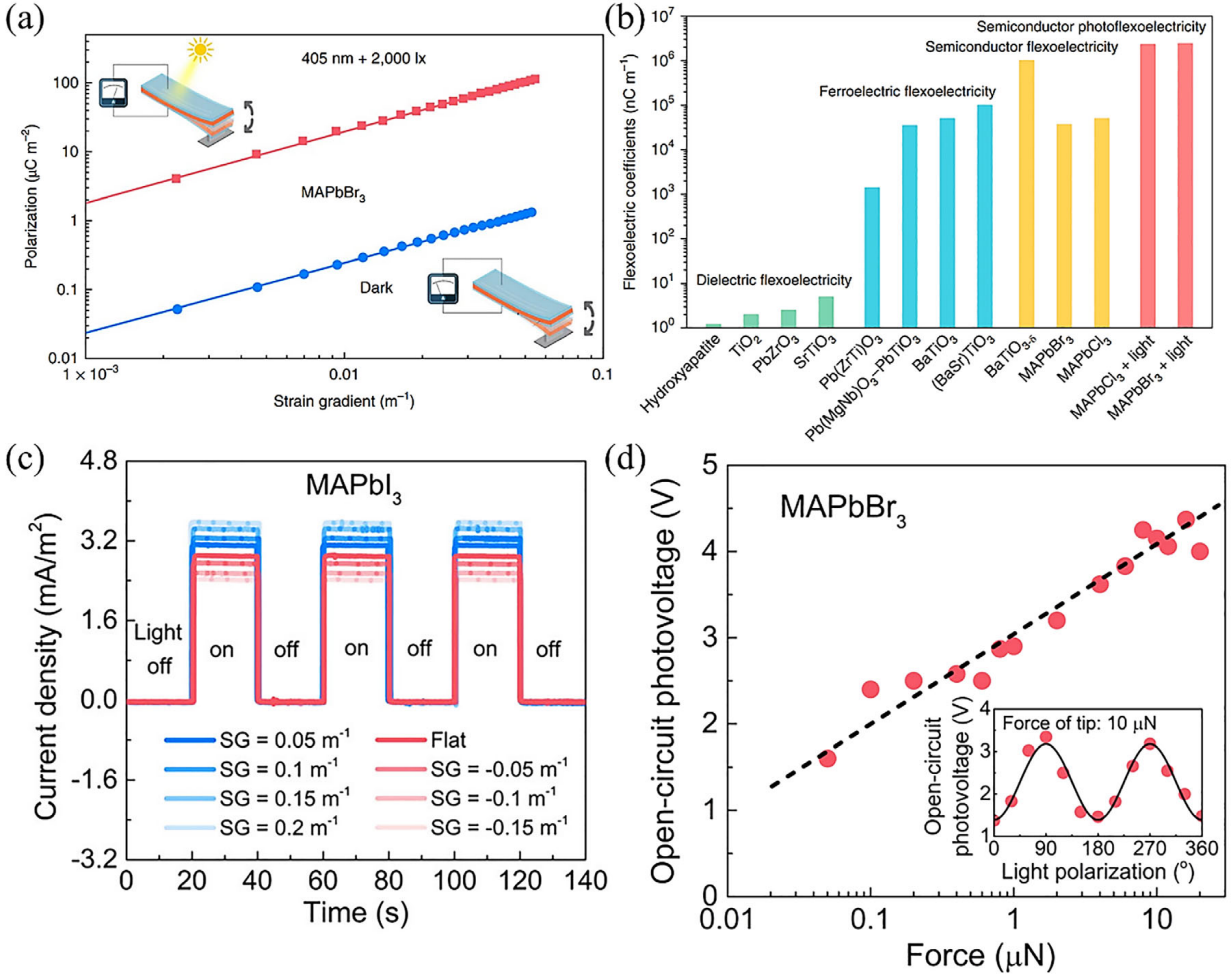
(a) Comparison between the bending‐induced polarization of MAPbBr3 in the dark and with light. (b) Comparison of effective flexoelectric coefficients for different materials. (c) Photovoltaic output of MAPbI_3_ under a series of strain gradients. (d) *V*_oc_–Force curve of MAPbBr_3_. (a,b) Adapted with permission from ref. [78] Copyright 2020 Springer Nature Limited. (c,d) Adapted with permission from ref. [98] Copyright 2024 American Physical Society.

The investigation of the flexo‐photovoltaic effect in halide perovskites was significantly advanced by Shu et al. [78], who demonstrated that, under simultaneous illumination and mechanical oscillation driven by a piezoelectric actuator, paraelectric hybrid organic–inorganic halide perovskites MAPbCl_3_ and MAPbBr_3_ (where MA denotes methylammonium, CH_3_NH_3_^+^) exhibit flexoelectric coefficients exceeding 2000 µC/m, orders of magnitude higher than values measured in the dark, as shown in Figure 14a. As summarized in Figure 14b, this record surpassed all other traditional ferroelectrics, even when an enhancement strategy was adopted. The authors argued that this enormous enhancement cannot be attributed solely to conventional bulk flexoelectricity, which scales with dielectric constant, nor to a flexoionic mechanism, which exhibits frequency dependence in the dark but vanishes under illumination. Instead, they correlated the phenomenon with barrier‐layer capacitor behavior, noting that halide perovskites exhibit significantly increased capacitance under illumination. This behavior is consistent with carrier‐enhanced flexoelectricity previously observed by the same group in oxygen‐vacancy‐doped BaTiO_3_ [77]. They defined a photo‐flexoelectric coefficient as *γ* = (*µ*_light_ − *µ*_dark_)/*µ*_dark_, reporting enhancement factors of up to 100‐fold, thereby highlighting the potential of such materials for photo‐electromechanical energy harvesting applications. In a follow‐up study, Wang et al. [98] quantified the flexo‐photovoltaic effect in single crystals of halide perovskites (MAPbBr_3_ and MAPbI_3_) and the benchmark oxide perovskite SrTiO_3_. The flexo‐photovoltaic response in halide perovskites was found to be orders of magnitude stronger than in SrTiO_3_, sufficient to generate photovoltages exceeding the material's bandgap. In MAPbI_3_, this effect coexists with a native, switchable ferroelectric‐like bulk photovoltaic response, as shown in Figure 14c. To quantify the flexo‐photovoltaic contribution, they introduced a flexo‐photovoltaic coefficient *Φ*_V_ = ∂*V*_oc_/∂*G*, where *V*_oc_ is the open‐circuit voltage under 1000 lux illumination, and *G* is the strain gradient. Linear regression yielded *Φ*_V_ = −3 × 10^−3^ V m for SrTiO_3_ and *Φ*_V_ = −1.3 V m for MAPbI_3_, reaching a nearly three‐order‐of‐magnitude enhancement under identical conditions. Using a conductive AFM tip to apply localized strain, they further showed that the open circuit photovoltage increases linearly with indentation force, surpassing the 2.3 eV bandgap to reach ∼4 V at ∼10 µN force. With a sharper tip, photovoltages up to ∼6 V, over twice the bandgap, were achieved even at lower forces, as demonstrated in Figure 14d.

Halide perovskite nanowires have demonstrated that the flexoelectric effect can not only be directly coupled with the photovoltaic effect but also show promising potential in photocatalytic and photoluminescence applications, highlighting its versatility in photo‐related energy conversion and light‐emission processes. Zhang et al. [171] demonstrated catalytic hydrogen production via photo‐mechanical coupling mediated by the photo‐flexoelectric effect in flexible methylammonium lead iodide (MAPbI_3_) nanowires suspended in hydrogen iodide solution. Under simultaneous light illumination and mechanical vibration, large strain gradients were generated within the nanowires, leading to a hydrogen production rate of 756.5 mmol/g/h. This rate surpasses those achieved by photocatalysis or flexocatalysis alone by nearly fivefold and fourfold, respectively. Pereira‐Andrade et al. [170] epitaxially grew CsPb(Br_1‐x_Cl_x_)_3_ nanowires and transferred them to fresh substrates for steady‐state and time‐resolved photoluminescence measurements. They characterized the photoluminescence intensity and lifetime across a temperature range spanning expected crystal phase transitions. The observed shifts in photoluminescence peak positions are attributed to flexoelectricity, which arises from strain gradients at ferroelastic domain walls that modulate electronic band edges, effectively creating a spatial variation in the electrochemical potential within the adiabatic approximation.

#### 3.2.2 AFM‐Tip‐Induced Local Strain Gradient

**FIGURE 15:**
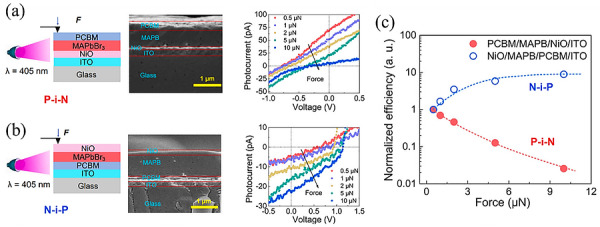
Schematic, SEM image, and photovoltaic output as a function of AFM force of (a) P‐i‐N and (b) N‐i‐P devices. (c) Normalized efficiency as a function of indentation force of N‐i‐P and P‐i‐N devices. Adapted with permission from ref. [172] Copyright 2025 Elsevier Inc.

The Shockley‐Queisser limit fundamentally constrains the maximum efficiency of conventional solar cell designs, whereas bulk photovoltaic mechanisms such as the flexo‐photovoltaic effect offer a pathway to surpass this barrier. Wang et al. [172] investigated strain gradient effects in halide perovskite solar cells and demonstrated that mechanical strain can significantly modulate their performance. When pressure was applied via an AFM tip approximately 1 µm from the illuminated edge, the photoelectric power, defined as the product of open‐circuit photovoltage and short‐circuit photocurrent, decreased in P‐I‐N devices but increased in N‐I‐P structured devices, as shown in Figure 15a,b. Although tip‐induced effects preclude precise absolute efficiency calculations, the authors quantified relative changes by normalizing the power output under indentation to that in the strain‐free state. This approach revealed an order‐of‐magnitude modulation in normalized efficiency, whose direction (enhancement or suppression) depends on whether the strain gradient aligns with or opposes the built‐in field of the device, as demonstrated in Figure 15c.

#### 3.2.3 Spontaneously Formed Strain Gradients

**FIGURE 16:**
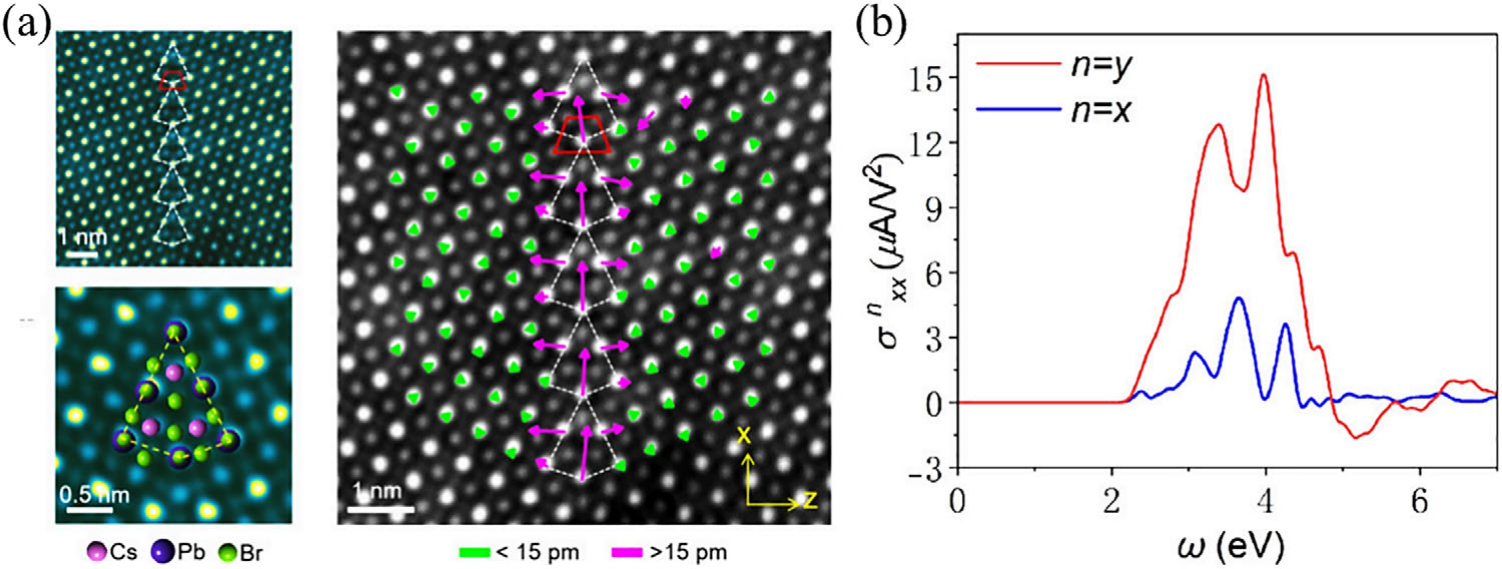
(a) HAADF‐STEM and corresponding iDPC images of atomic‐scale displacements and strain gradients at the 52° grain boundary of CsPbBr_2_Cl. (b) Calculated effective shift‐current spectra as a function of optical frequency ω upon illumination parallel to the grain boundary, where *n* = *x* and *n* = *y* represent the photocurrent along the vertical and parallel grain boundary direction, respectively. Adapted with permission from ref. [92] Copyright 2025 American Chemical Society.

Grain boundaries, commonly present in halide perovskites due to substrate mismatch, structural distortions, or compositional segregation, significantly influence their optoelectronic performance. These interfaces introduce substantial strain and composition gradients, activating flexoelectric effects that can enhance photovoltaic behavior. Xiong et al. [92] utilized aberration‐corrected transmission electron microscopy to directly resolve the atomic structure of grain boundaries, revealing the emergence of flexoelectric polarization and corresponding shift currents. Their results show that a 52° grain boundary produces a pronounced strain gradient, leading to strong flexoelectric polarization, a phenomenon consistently observed across boundaries with varying compositions and misorientation angles, as shown in Figure 16a. As shown in Figure 16b, first‐principles calculations further confirm that such flexoelectric polarization enhances the photovoltaic response, generating a shift current of approximately 15 µA/V^2^.

### 3.3 Section Summary

Research on flexoelectric effects in halide perovskites has expanded from fundamental studies of flexo‐photovoltaic phenomena to a broad range of photoconversion applications. Early works established that strain gradients can induce giant flexo‐photovoltaic responses, yielding photovoltages that exceed the bandgap in materials such as MAPbI_3_ and MAPbBr_3_. Subsequent studies demonstrated the critical role of flexoelectricity in enhancing photocatalytic hydrogen production and in modulating photoluminescence behavior, highlighting its multifunctional potential. Mechanistically, strain gradients introduced via bending, AFM indentation, or phase‐transition‐coupled substrates break inversion symmetry and promote efficient carrier separation. Notably, nanowire and freestanding configurations have enabled pronounced strain–light coupling, further extending applications toward adaptive and energy‐harvesting devices. Despite these advances, key challenges remain, including the decoupling of surface and bulk flexoelectric contributions and the realization of stable, scalable strain engineering over large areas. Overall, halide perovskites exemplify how flexoelectricity can be harnessed not only for enhanced photovoltaic performance but also for tunable photoemission and catalysis, positioning them as a versatile platform for next‐generation opto‐mechano‐electronic systems.

## 4 Flexo‐Photoconversion in 2D Materials

### 4.1 2D Materials and Their Features

The history of 2D materials, once considered thermodynamically unstable, was fundamentally rewritten with the ground‐breaking isolation of graphene in 2004 by Andre Geim and Konstantin Novoselov [173]. They used a simple mechanical exfoliation technique (“scotch‐tape method”) to peel atomically thin layers from a graphite crystal, revealing a host of extraordinary properties and earning them the Nobel Prize in Physics in 2010. This discovery ignited a global research effort that rapidly expanded beyond graphene. The field broadened to include a vast family of other 2D materials, often referred to as the “2D crystal library,” which includes insulators like hexagonal boron nitride (h‐BN), semiconductors such as transition metal dichalcogenides (TMDs, e.g., MoS_2_ and WSe_2_), and even superconductors and topological insulators. The rapid expansion has been fueled by the development of various synthesis methods, from chemical vapor deposition (CVD) for large‐area films to liquid‐phase exfoliation for mass production.

**FIGURE 17:**
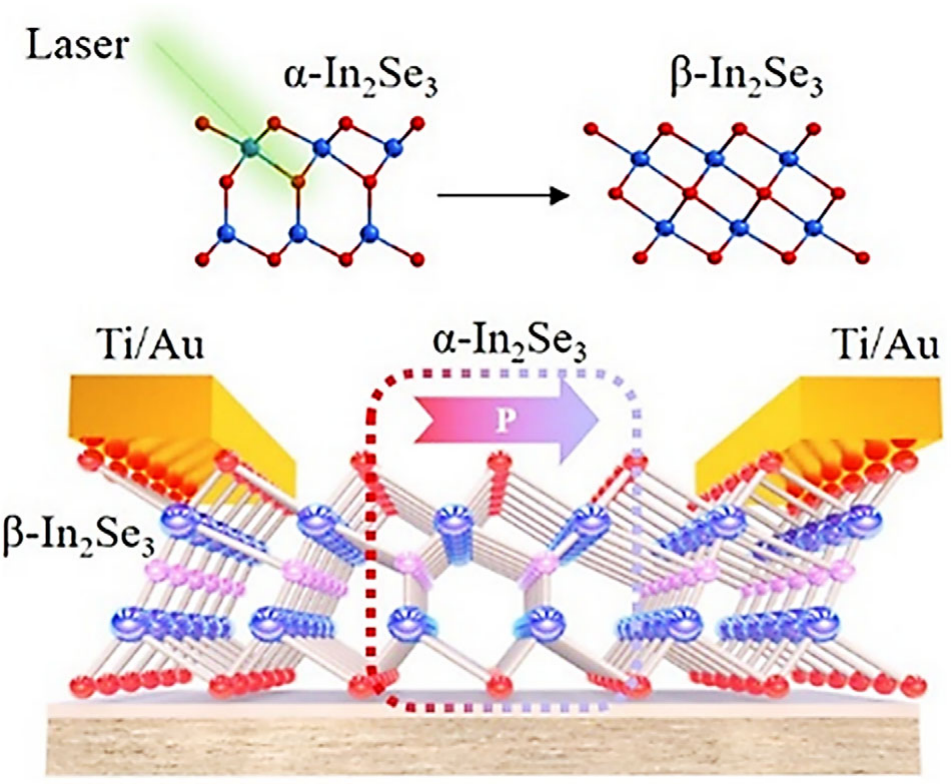
Structure of 2D *α*‐ and *β*‐In_2_Se_3_ monolayer. Adapted with permission from ref. [174] Copyright. 2022 American Chemical Society.

Despite their diverse chemical compositions and electronic properties, all 2D materials share a common defining structural feature: the atomically thin, layered architecture, as shown in Figure 17 (using *α*‐ and *β*‐In_2_Se_3_ monolayers as the representative example [174]). The inherent structural flexibility of low‐dimensional materials can complicate the identification of the fundamental origins of their physical properties. In the case of ferroelectricity in 2D systems, intrinsic contributions arise from a combination of electronic and ionic effects, along with lattice distortion. Extrinsic ferroelectricity, meanwhile, can be induced through various external means, such as strain, charge doping, defect engineering, interface modification, surface functionalization, and boundary conditioning. These approaches allow deliberate alteration of electronic or lattice structures, thereby creating opportunities to break intrinsic symmetry and engineer emergent ferroelectric responses [175, 176]. In their bulk form, these materials are composed of strong in‐plane covalent bonds within each layer, which are stacked together by weak out‐of‐plane van der Waals forces. This unique bonding structure gives rise to several universal and important physical properties. These include quantum confinement [177, 178, 179, 180], which tailors their electronic band structure (e.g., making graphene a semi‐metal and MoS_2_ a direct‐bandgap semiconductor when thinned to a monolayer), and high surface‐to‐volume ratio, which makes them extremely sensitive to the environment. Furthermore, their layered nature allows for “van der Waals heterostructures,” where different 2D materials can be stacked like Lego blocks to create artificial solids with customized properties [181, 182, 183, 184, 185, 186, 187, 188, 189]. These remarkable characteristics have led to a wide range of application examples, from ultra‐fast and flexible transistors and high‐sensitivity sensors to novel optoelectronic devices like photodetectors and LEDs, and even advanced energy storage and catalysis systems.

Two‐dimensional materials present a uniquely advantageous platform for studying and harnessing the flexoelectric effect, primarily due to their extreme mechanical properties and atomic‐scale thickness. The key lies in the fundamental equation for flexoelectric polarization. In any bent or deformed material, the strain gradient is inversely proportional to the thickness. Consequently, atomically thin 2D materials, when subjected to bending or rippling, can experience colossal strain gradients that are orders of magnitude larger than what is achievable in bulk solids [190, 191, 192, 193, 194]. This enables the generation of exceptionally strong and highly tunable flexoelectric fields from even minimal mechanical deformations. Furthermore, the absence of dangling bonds and their high mechanical strength allow them to sustain large elastic strains without fracture, making them ideal for creating strain‐engineered devices.

In the context of photovoltaics, 2D materials, particularly semiconducting TMDs like MoS_2_ and WSe_2_, offer a compelling set of advantages that dovetail perfectly with flexoelectricity [23, 195, 196, 197, 198, 199, 200]. From a preparation standpoint, their layered nature allows for mechanical exfoliation for fundamental studies and scalable CVD for device fabrication, facilitating the creation of complex van der Waals heterostructures without lattice‐matching constraints. Crucially, their bandgap is highly tunable [201, 202, 203, 204, 205]; TMDs undergo a transition from an indirect bandgap in the bulk to a direct bandgap in the monolayer, providing strong light‐matter interaction and a bandgap ideally suited for efficient solar light absorption (e.g., ∼1.8 eV for monolayer MoS_2_). The combination of these properties creates a powerful synergy: the giant flexoelectric field in a bent 2D TMD can act as a built‐in, non‐uniform electric field that efficiently separates photogenerated electron–hole pairs across the entire material. This flexo‐photovoltaic coupling can significantly enhance the performance of a 2D solar cell by boosting both the open‐circuit voltage and the short‐circuit current, offering a novel pathway to overcome recombination losses and design high‐efficiency, mechanically responsive optoelectronic devices.

The compelling advantages of 2D materials, particularly their propensity for generating giant strain gradients and their tunable optoelectronic properties, establish a powerful foundation for exploring flexo‐photovoltaic coupling [23, 190, 191, 192, 193, 194, 195, 196, 197, 198, 199, 200, 206]. Having established this conceptual framework, we now transition to a detailed analysis of experimental literature. The following section will critically examine seminal works that have directly demonstrated and quantified this effect, beginning with pioneering studies on strain‐engineered MoS_2_ and WSe_2_ monolayers, and progressing to more complex van der Waals heterostructures designed specifically to harness flexoelectric polarization for enhanced photovoltaic performance.

### 4.2 Progress, Strategies, and Mechanisms

Given their inherent flexibility, researchers have naturally developed two primary approaches for applying strain gradients to 2D materials: placement on an uneven (pre‐patterned or curved) substrate, and suspension across gaps or trenches. In addition, AFM‐based nanoindentation remains a widely used method both for applying localized high strain gradients and for simultaneous electrical characterization. Accordingly, the studies reviewed in this section are grouped into three categories based on the strain‐gradient application strategy: (a) Suspended membrane deformation, (b) Uneven substrate bending, and (c) AFM‐tip‐induced local deformation. The magnitude, repeatability, and scalability of strain gradient induction methods are listed in Table 7.

**TABLE 7:** Magnitude, repeatability, and scalability of various strain gradient induction methods in 2D materials.

Source of strain gradient	Magnitude (m^−1^)	Repeatability	Scalability
Suspended deformation	10^4^–10^6^	Low	Low
Uneven substrates	10^−2^–10^1^	High	High
AFM probe pressing	10^7^	Low–moderate	Very low

For 2D materials, strain gradients have been primarily generated through three approaches. Suspended structures (e.g., bubbles or airbridges) enable ultrahigh strain gradients in a clean, substrate‐decoupled environment, offering moderate repeatability but poor scalability due to fragile membranes and complex fabrication. Non‐flat substrates (e.g., nanopillars or trenches) provide excellent repeatability and scalability, allowing wafer‐scale arrays with uniform strain gradients, albeit at the cost of interfacial interference from the underlying substrate. AFM tip loading allows localized and reversible strain gradient engineering with low repeatability and very low scalability, making it suitable for fundamental mechanism studies rather than practical device fabrication. Strengths and drawbacks are shown in Table 8, and related works are summarized in Table 9.

**TABLE 8:** Strengths and drawbacks of various strain gradient induction methods in 2D materials.

Source of strain gradient	Strengths	Drawbacks
Suspended deformation	Substrate‐decoupled compatible with in situ techniques	Mechanical instability poor uniformity
Uneven substrates	High reproducibility robust mechanical support	Strong substrate interference hard to decouple from strain
AFM probe pressing	Large localized strain gradient in situ local measurement	Downward strain gradient only only applied to the surface area

**TABLE 9:** Summary of flexo‐photoconversion studies in 2D materials.

Source of strain gradient	Material system	Properties/phenomenon	Ref
Suspended deformation ∼10^4^ m^−1^	InSe, WSe_2_	Light‐induced surface potential regulation from −1.2 to 0.3 V.	[207]
Suspended deformation and AFM probe pressing ∼10^6^ m^−1^	2H‐MoS_2_	A 41‐fold *I*_sc_, a responsivity of 191 mA/W, and a photoconductance of multilevel achieved	[208]
Suspended deformation and AFM probe pressing ∼10^6^ m^−1^	CIPS	A 20‐fold *I*_sc_, a responsivity of 245 mA/W, and a detectivity of 1.73 × 10^11^ Jones achieved	[99]
Suspended deformation DFT simulation	CrSBr monolayer	*J*_sc_ of 4.6 A/m^2^ and photo response of 36 mA/W obtained	[209]
Uneven substrate bending ∼10^6^ m^−1^	MoS_2_	*I*_sc_ is orders of magnitude enhanced around the strain‐gradient area, and record‐high BPV coefficients are achieved	[96]
Uneven substrate bending ∼10^6^ m^−1^	α‐In_2_Se_3_/β‐InSe heterojunction	A 2.48‐fold *V*_oc_ and a 7.62‐fold zero‐biased responsivity were achieved	[194]
Uneven substrate bending	Violet phosphorene nanosheets	A BPV coefficient of 1.3 × 10^−3^/V and a polarization extinction ratio of 21.6 (typically less than 3) achieved	[210]
Pre‐curved substrate growth ∼50 m^−1^	Bi_2_Te_3_	Output voltage is enhanced by 15% under the concave condition and decreased under the convex condition	[211]
AFM probe pressing ∼10^7^ m^−1^	graphene–silicon Schottky junction	*V*_oc_ enhanced from 0.38 to 0.46 V, and power conversion efficiency enhanced by 20%.	[212]
AFM probe pressing ∼10^7^ m^−1^	α‐MoO_3_	Photocurrent increased from 9.8 to 17.2 nA as the applied stress increasing	[100]

#### 4.2.1 Suspended Membrane Deformation

**FIGURE 18:**
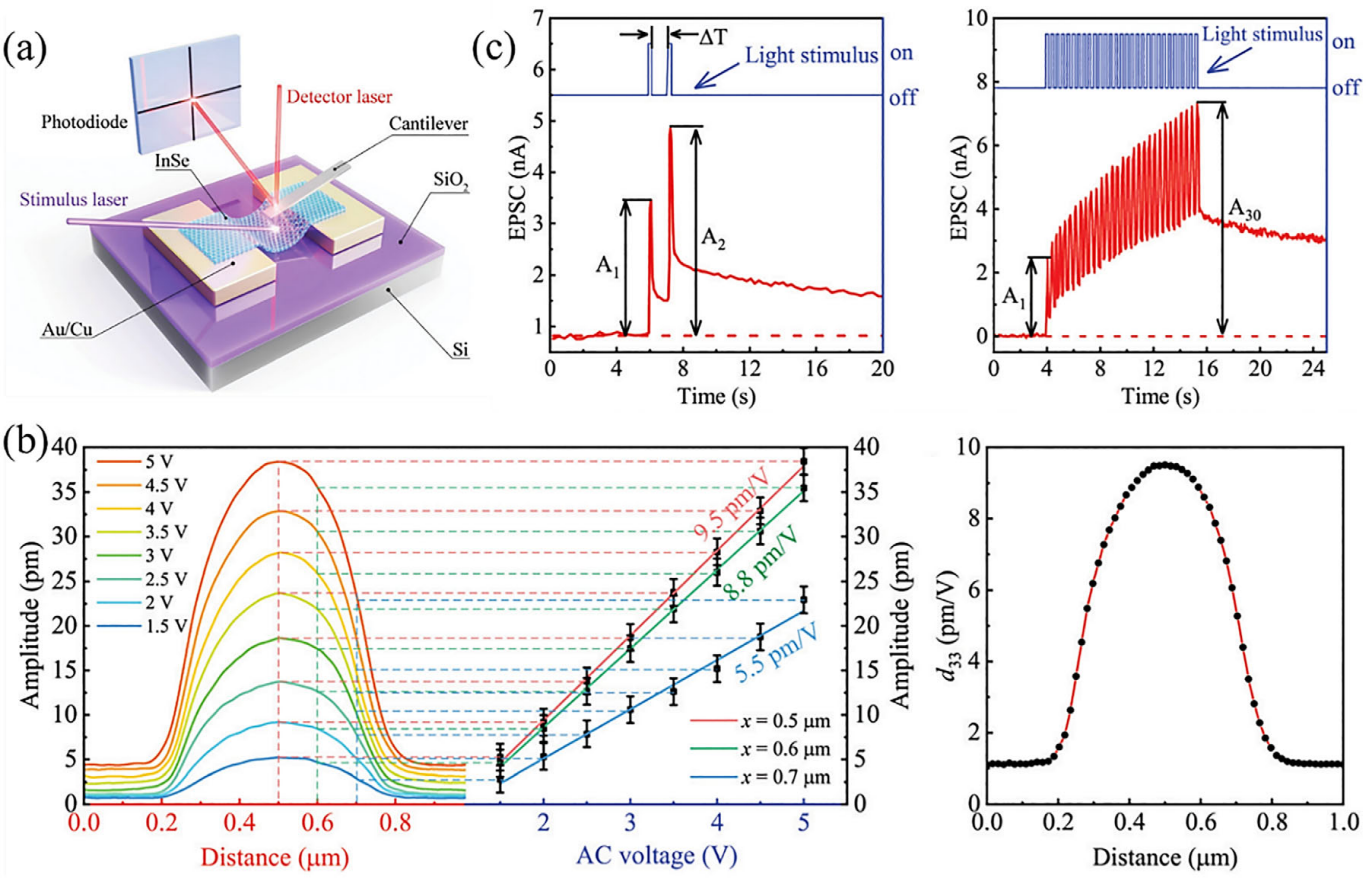
(a) Experimental schematic of PFM and lighting‐KPFM system on the bent InSe device structure. (b) Statistical distribution of PFM amplitude–displacement and effective piezoelectric coefficient–voltage curves. (c) Pair‐pulse facilitation (PPF) and excitatory post‐synaptic current (EPSC) behaviors of InSe artificial synapses. Adapted with permission from ref. [207] Copyright 2023 The Royal Society of Chemistry.

Wang et al. [207] provided the first direct nanoscale experimental evidence of coupling between flexoelectric and photoelectric effects through coordinated manipulation of light and strain gradients. Using high‐resolution piezoresponse force microscopy, they mapped the flexoelectric polarization induced by strain gradients in bent 2D InSe and WSe_2_ flakes, as shown in Figure 18a. Figure 18b demonstrates that the measured effective out‐of‐plane piezoelectric coefficient *d* ^eff^_33_ reached maximum values of approximately 9.5 and 4.9 pm/V at the centers of bent InSe and WSe_2_ channels, respectively, decreasing toward both edges, which consistent with a gradient distribution of flexoelectric polarization. In situ Kelvin probe force microscopy further revealed the corresponding redistribution of charge carriers, showing that back‐to‐back built‐in electric fields formed in the bent semiconductors, promoting separation of photogenerated electron–hole pairs and trapping carriers at the channel centers. Under illumination, the trapped carriers were slowly released, leading to a sustained photoconductance effect. Based on this mechanism, the authors designed an artificial synapse using bent 2D InSe, successfully emulating neurosynaptic behaviors such as short‐term plasticity, long‐term plasticity, and high‐pass filtering, as indicated in Figure 18c.

**FIGURE 19:**
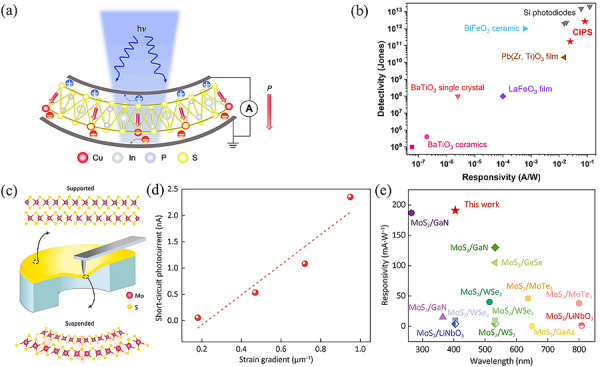
(a) Schematics of bulk photovoltaic effect in the 2D ferroelectric CIPS in flexoelectric engineered state. (b) Comparison of detectivity versus responsivity of CIPS photodetector with other ferroelectric‐based photodetectors and commercial Si photodiodes. (c) Side‐view schematics of the 2H‐MoS_2_ lattice structure in the substrate‐supported and suspended regions. (d) Zero‐bias photocurrent in suspended 2H‐MoS_2_ as a function of the strain gradient. (e) Compare the self‐powered responsivity of MoS_2_‐based photodetectors. (a,b) Adapted with permission from ref. [99] Copyright 2024 American Chemical Society. (c‐e) Adapted with permission from ref. [208] Copyright 2025 The Chinese Ceramic Society Published by Elsevier B.V. .

Yu et al. [99] achieved active mechanical control of the BPV in suspended 2D CuInP_2_S_6_ (CIPS), a ferroelectric material with high susceptibility to external fields, as shown in Figure 19a. The suspended CIPS structure exhibited a 20‐fold enhancement in photocurrent, which could be continuously tuned through either applied mechanical force or light polarization orientation. Figure 19b summarizes that the resulting flexoelectrically engineered photodetector, actuated by air pressure and without further optimization, demonstrated a responsivity of 2.45 × 10^−2^ A/W and a detectivity of 1.73 × 10^11^ Jones, surpassing typical ferroelectric‐based photodetectors and approaching those of commercial silicon photodiodes. In another work, Yu et al. [208] introduced strain gradients into suspended 2H‐MoS_2_, breaking its intrinsic inversion symmetry and inducing a BPV in the otherwise centrosymmetric material, as demonstrated in Figure 19c. Figure 19d indicates that the resulting flexoelectric polarization promotes efficient separation of photogenerated carriers, yielding a 41‐fold enhancement in short‐circuit photocurrent under a strain gradient of 0.95/µm. The flexoelectric modulated photodetector allows dynamic tuning via air pressure, enabling multilevel photoconductance states and achieving a responsivity of 191 mA/W. This performance exceeds that of previously reported self‐powered MoS_2_‐based photodetectors, establishing a viable strategy for high‐performance optoelectronic devices, as shown in Figure 19e. Chen et al. [209] demonstrated that mechanical bending of monolayer CrSBr enables effective spatial separation of photogenerated electrons and holes. The strain gradient modulates both shift and ballistic current densities in non‐equilibrium carrier transport, inducing a pronounced photovoltaic response. Concurrently, the flexoelectric field establishes a built‐in electric field that promotes self‐powered device operation. Furthermore, the resulting polarized photodetector exhibits excellent spin‐dependent photovoltaic characteristics and high polarization sensitivity.

#### 4.2.2 Uneven Substrate Bending

**FIGURE 20:**
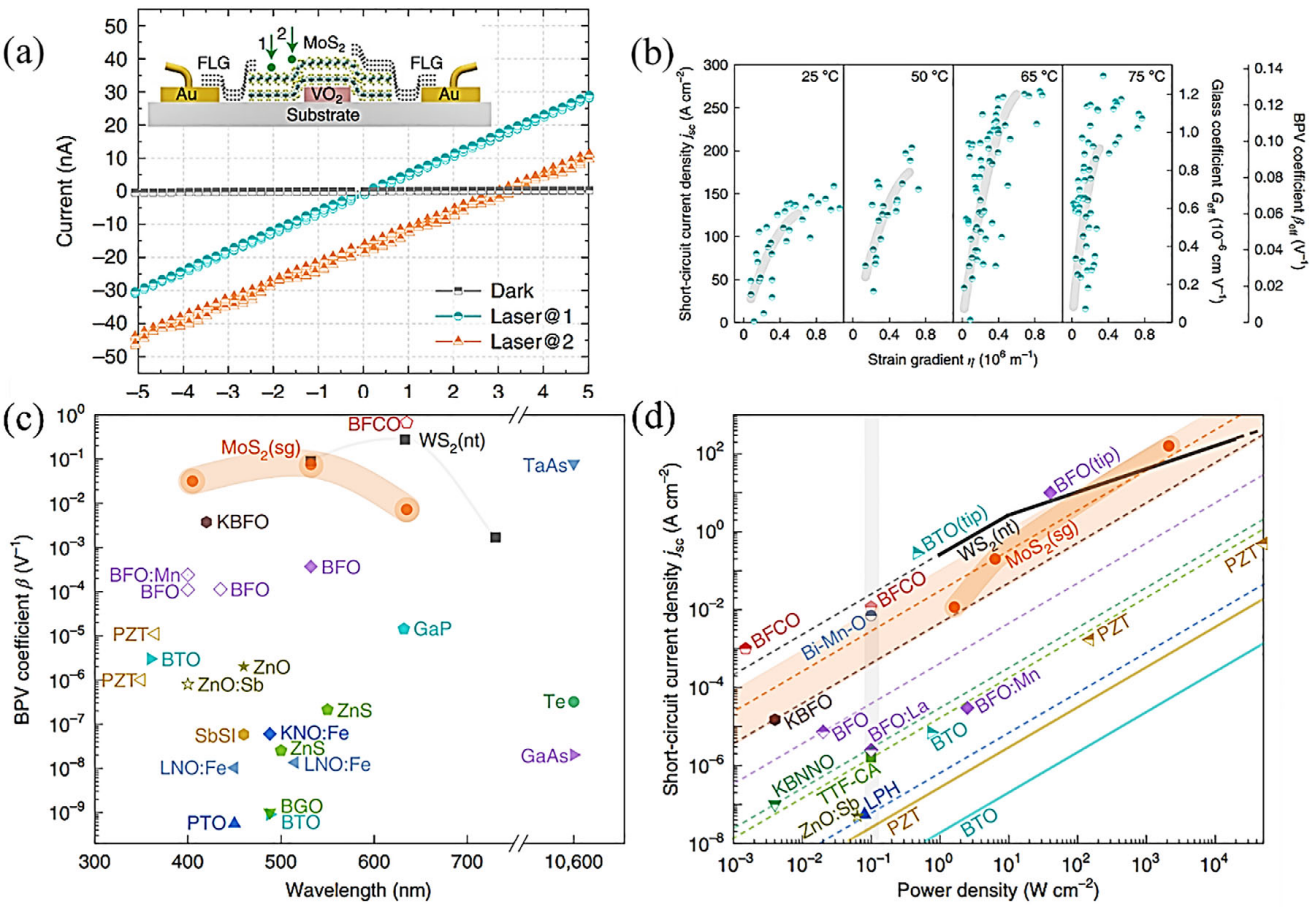
(a) Current‒voltage curves of the device under 532 nm laser illumination and without illumination. (b) Plots of short‐circuit current density *J*_sc_ versus strain gradient *η*. (c) Experimental BPV coefficients *β* for non‐centrosymmetric materials. (d) Short‐circuit current density *J*_sc_ versus incident power density in reported non‐centrosymmetric materials. Adapted with permission from ref. [96] Copyright 2021 Springer Nature Limited.

Jiang et al. [96] experimentally demonstrated the flexo‐photovoltaic effect in the archetypal 2D material MoS_2_ using a strain‐gradient engineering approach based on structural inhomogeneity and phase transition in a MoS_2_/VO_2_ hybrid system, as shown in Figure 20a. During the temperature‐induced structural phase transition of the VO_2_ microbeam, the MoS_2_ sheet supported giant strain gradients up to 10^6^ m^−1^. As shown in Figure 20b, the observed enhancement in both strain gradient and photocurrent near the phase transition temperature confirms the effectiveness of this mechanism in modulating the flexo‐photovoltaic response. The authors quantified the phenomenon using the Glass coefficient (*G*) and bulk photovoltaic coefficient (*β*), derived from measured photocurrents. Results indicate that the effective bulk photovoltaic coefficient in strain‐engineered MoS_2_ reaches values orders of magnitude higher than those observed in most non‐centrosymmetric materials, as demonstrated in Figure 20c. Such material also has exceptional properties in terms of short circuit current density and power density ratio, as shown in Figure 20d.

**FIGURE 21:**
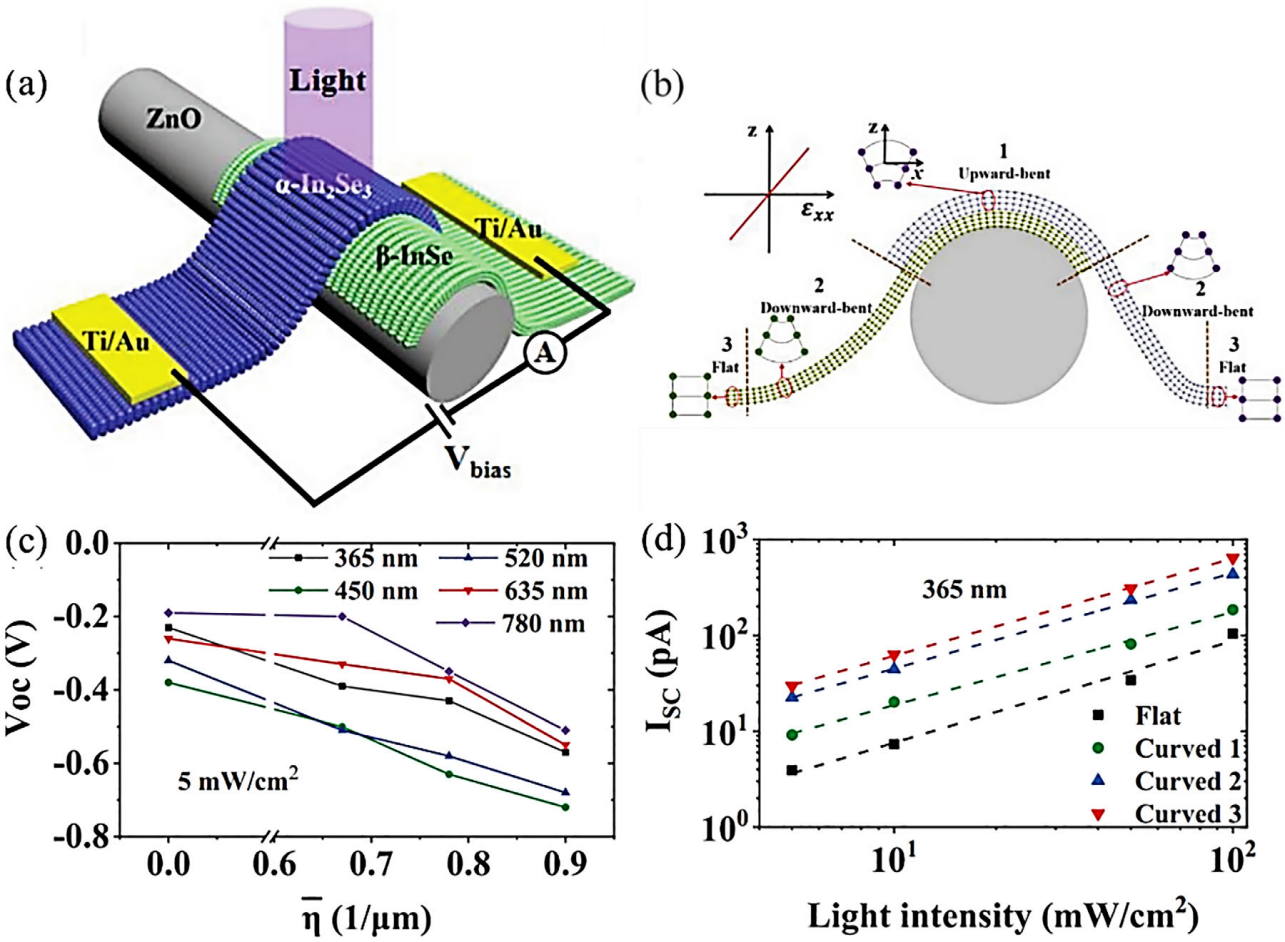
(a) Schematic diagram of the curved 𝛼‐In2Se3/𝛽‐InSe heterojunction. (b) Schematic diagram of the different curved states in the heterojunctions: upward‐bent state, downward‐bent state, and flat state. (c) Dependency of *V*_oc_ on the curvature under 5 mW/cm^2^ light illuminations with different wavelengths. (d) Dependency of *I*_sc_ on the power intensities under 365 nm light illumination. Adapted with permission from ref. [194] Copyright 2024 Wiley‐VCH GmbH.

Qi et al. [194] fabricated curved *α*‐In_2_Se_3_/*β*‐InSe heterojunctions by bending them over nanowires of varying diameters, achieving large curvatures of 0.1‐1 µm^−1^, as shown in Figure 21a,b. Kelvin probe force microscopy confirmed significant band alignment modulation in α‐In_2_Se_3_, resulting from the bending‐induced flexoelectric effect. The strain‐induced piezoelectric contribution was negligible due to weak van der Waals interfacial coupling, while flexoelectric polarization in *β*‐InSe was effectively screened by electron accumulation within the unilaterally depleted heterojunction. As shown in Figure 21c,d, compared to a flat heterojunction, the curved structure with an average curvature of 0.9/µm exhibited 2.48‐fold and 7.62‐fold enhancements in open‐circuit voltage and zero‐bias responsivity, respectively.

Sun et al. [210] induced a pronounced flexo‐photovoltaic effect in strained violet phosphorene nanosheets by implementing strain engineering at hexagonal boron nitride nanoedges, marking the first observation of this phenomenon in a non‐TMD system. The bulk photovoltaic response originates from the breaking of inversion symmetry due to uniaxial strain imposed on the phosphorene at the h‐BN edge. Through thickness‐dependent photovoltage measurements, the study established a direct correlation between the BPV and strain gradients in the material. By systematically optimizing both the h‐BN nanoedge height and the phosphorene thickness, the achieved bulk photovoltaic coefficient reached 1.3 × 10^−3^/V with a polarization extinction ratio of 21.6, which exceeds those reported for most TMD systems. The flexo‐photovoltaic effect is also gaining attention in the infrared spectral region. Takada et al. [211] deposited Bi_2_Te_3_ thin films onto pre‐curved flexible substrates, inducing strain upon flattening the films. The pre‐curvature during deposition was found to influence both crystal orientation and strain state. The Bi_2_Te_3_ film prepared under a concave pre‐curved condition exhibited an output voltage approximately 15% higher than that of a film deposited on a flat substrate.

#### 4.2.3 AFM‐Probe‐Induced Local Strain Gradients

**FIGURE 22:**
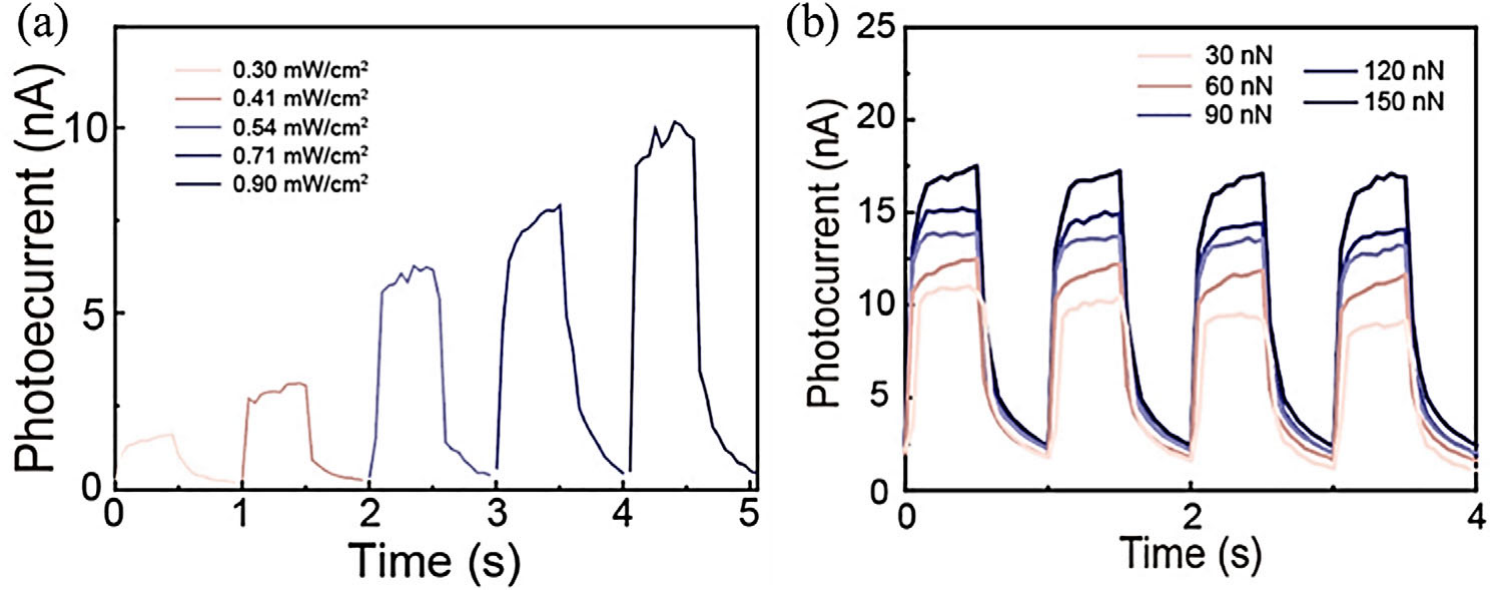
(a) Photocurrent response at various power densities under 365 nm and 5 V driving voltage. (b) The photocurrent response under C‐AFM probe pressing of 30–150 nN. Adapted with permission from ref. [100] Copyright 2025 Elsevier Ltd.

Hu et al. [100] investigated the flexo‐photovoltaic properties of 2D *α*‐MoO_3_ nanocrystals synthesized via CVD. The material crystallizes in the centrosymmetric *Pbnm* space group, yet piezo‐response force microscopy amplitude and phase mappings revealed a pronounced flexoelectric response, confirming the existence of a converse flexoelectric effect. A dual‐electrode photodetector was fabricated to characterize the photo‐response, showing that the output photocurrent increased continuously with laser power density (0.3‐0.9 mW/cm^2^) and bias voltage (1‐5 V), reaching a maximum of ∼9.8 nA at 0.9 mW/cm^2^ and 5 V, as shown in Figure 22a. Notably, from Figure 22b, the photocurrent exhibited a clear stress‐dependent enhancement, rising from 9.8 to 17.2 nA as the applied stress increased from 30 to 150 nN, demonstrating effective strain‐gradient modulation of the photovoltaic behavior. The stress‐dependent current enhancement can be attributed to flexoelectric modulation of electron transport, and the relation can be described by classical thermal emission theory.

The graphene‐silicon Schottky junction (GSJ), with its potential for large‐scale fabrication and integration, offers a promising platform for Schottky solar cells. However, its PCE is fundamentally limited by a low open‐circuit voltage *V*_oc_, which is constrained by the Schottky barrier height. Pu et al. [212] introduced an electromechanical approach based on the flexoelectric effect to enhance the photovoltaic performance of GSJ devices. Through AFM tip‐induced indentation and simultaneous current‐voltage (*I‐V*) characterization under controlled strain gradients, they observed a maximum 20% increase in *V*_oc_, resulting in a clear enhancement of the overall PCE.

### 4.3 Section Summary

Research on flexo‐photovoltaic effects in 2D materials has advanced through several distinct experimental routes, which can be broadly classified according to the mode of strain application: (1) substrate‐mediated bending (e.g., pre‐curved or patterned supports), (2) suspended membrane deformation, and (3) AFM‐tip‐induced local straining. These approaches have demonstrated that well‐controlled strain gradients can break inversion symmetry in otherwise centrosymmetric 2D systems such as monolayer MoS_2_, WSe_2_, and phosphorene, thereby enabling BPVs and pronounced photocurrent enhancement. Key advances include the achievement of strain‐tunable photovoltages exceeding the bandgap, the design of flexoelectrically modulated artificial synaptic devices, and the demonstration of mechanically reconfigurable photodetectors with enhanced responsivity and detectivity. Furthermore, in hybrid heterostructures combining 2D semiconductors with phase‐change materials (e.g., VO_2_) or ferroelectrics (e.g., CuInP_2_S_6_), strain gradients have been shown to dynamically tailor band alignment and charge carrier dynamics. Collectively, these studies underscore the potential of 2D materials as versatile platforms for adaptive, strain‐engineered optoelectronics. At the same time, they underscore the critical need for scalable, controllable, and reproducible strain‐integration strategies to facilitate the transition from proof‐of‐concept demonstrations to practical device applications.

## 5 Challenges, Future Perspectives, and Summary

**FIGURE 23:**
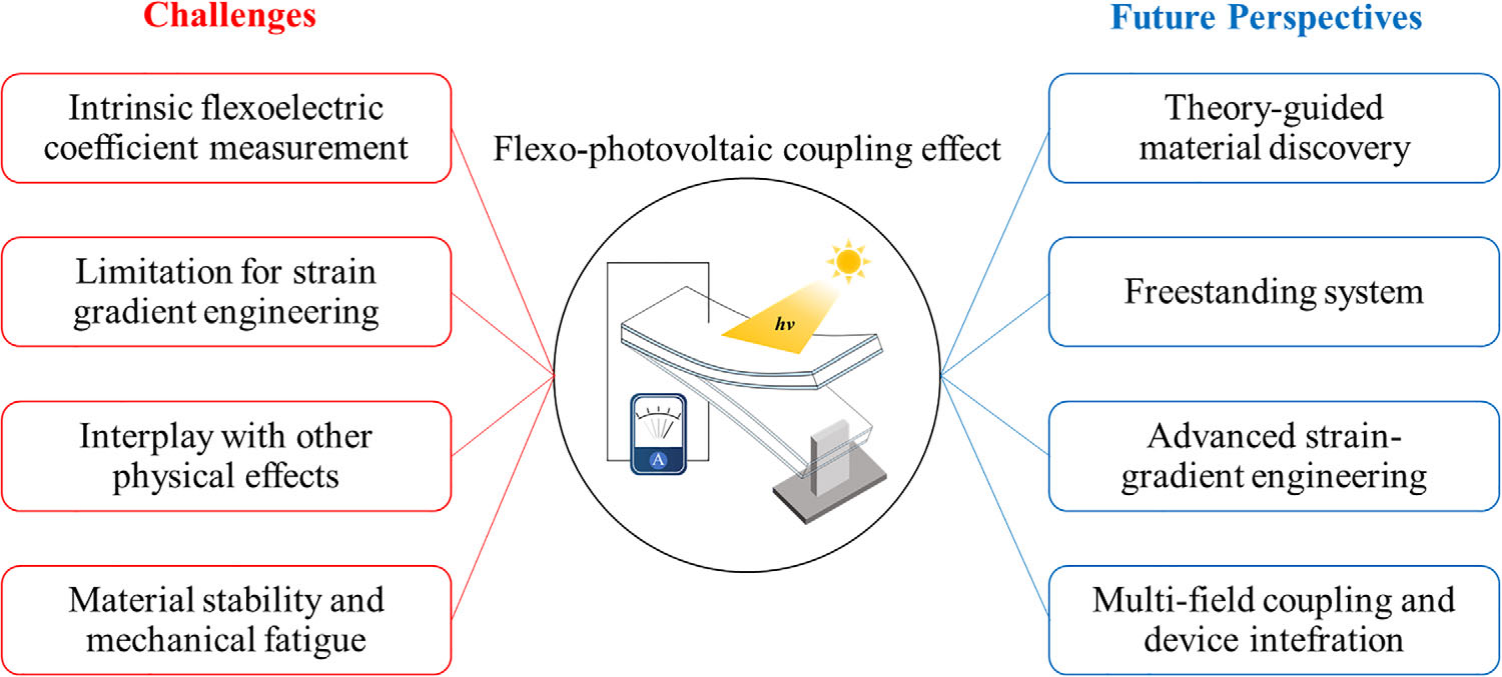
Schematic diagram of the challenges and future perspectives of flexo‐photovoltaic coupling effect.

Despite the considerable progress outlined in previous sections, the development of practical flexo‐photovoltaic devices faces several persistent challenges in materials, characterization, and integration. This section synthesizes these key limitations and outlines corresponding future research directions needed to advance the field toward functional applications, as shown in Figure 23.

### 5.1 Current Challenges

Despite the rapid progress in understanding and harnessing the flexo‐photovoltaic effect across diverse material systems, several fundamental and practical challenges still remain to be addressed before the full potential can be realized in functional energy‐harvesting and optoelectronic devices. The main challenges related to the flexo‐photovoltaic effect and its application in energy and optoelectronic device applications are summarized as follows:

a. Quantification and Isolation of the Intrinsic Flexoelectric Coefficient

A major challenge lies in the accurate quantification of the intrinsic flexoelectric coefficient (*µ*), particularly in emerging material systems such as halide perovskites and 2D semiconductors. As discussed in ref. [78], experimentally measured values often correspond to effective flexoelectric coefficients that conflate bulk flexoelectricity with surface contributions, ion migration, and photoconductive effects. In materials like MAPbI_3_, where mobile ionic species and interfacial phenomena are pronounced, decoupling these intertwined factors remains nontrivial. Therefore, one can only assume that the magnitude of order is appropriate [98]. The lack of standardized measurement protocols further complicates direct comparison between different studies and material classes.

b. Limitations of Epitaxial Thin Films for Strain Engineering

Epitaxially grown thin films, while offering high crystalline quality and strong strain coupling, face inherent limitations in exploiting the flexo‐photovoltaic effect. Their mechanical rigidity, imposed by the underlying stiff substrate, severely limits the magnitude of applicable macroscopic strain gradient or curvature, which would often be limited within 10^−1^ m^−1^ [90]. In other cases, lattice mismatch strain relaxation indeed generates local strain gradient over 10^6^ m^−1^ [139, 140, 141], but such a strain gradient cannot be withdrawn, let alone tunable. Furthermore, the epitaxial growth process hinders the transfer of active layers onto flexible or unconventional substrates, thereby restricting optical design flexibility, such as angle‐dependent or substrate‐transparent illumination, and complicating integration into advanced or flexible device architectures.

c. Interplay With Other Physical Effects

The flexo‐photovoltaic response frequently coexists and interacts with other physical mechanisms such as ferroelectric polarization, piezoelectricity, pyroelectricity, and ionic conduction (e.g., flexoionic effects). In materials like BFO or CIPS [90, 99, 141], disentangling the respective contributions of these effects to the overall photocurrent remains challenging yet is critical for designing the optimized device architectures. Moreover, in semiconductors with strong light–matter coupling, photocarrier dynamics and band‐structure renormalization under strain gradients further complicate predictive modeling and performance optimization.

d. Material Stability and Mechanical Fatigue

Many experimental demonstrations of the flexo‐photovoltaic effect rely on mechanically bending or straining materials, especially in flexible or freestanding configurations. Repeated mechanical cycling can induce microcracks, phase segregation, or interfacial degradation. These issues are particularly pronounced in hybrid perovskites and oxide heterostructures, as some researchers have attempted to solve [66]. The long‐term stability of flexo‐photovoltaic devices under sustained or cyclic strain remains largely unexplored, posing a significant hurdle for practical applications such as wearable energy harvesters or adaptive optoelectronic systems.

### 5.2 Future Perspectives

Looking ahead, several promising directions are emerging to address the aforementioned challenges, among which the development of freestanding thin films stands out as a particularly pivotal strategy.

a. Theory‐Guided Material Discovery

Combining high‐throughput computational screening and machine learning to systematically identify new materials with large intrinsic flexoelectric coefficients, high charge carrier mobility, and suitable bandgaps, such as 2D ferroelectrics, layered perovskites, or topological materials, could populate a materials genome for next‐generation flexo‐photovoltaic devices. Simultaneously, developing multiscale theoretical models that accurately describe non‐equilibrium carrier dynamics under strain gradients will be essential for establishing predictive design rules and guiding the device optimization [213, 214].

b. Freestanding Films as a Versatile Platform

Releasing high‐quality epitaxial films from their rigid substrates to form freestanding membranes could fundamentally overcome the limitations of conventional epitaxial systems. This approach not only unlocks mechanical flexibility by enabling the application of larger and more uniform macroscopic strains but also allows transfer onto arbitrary flexible or curved substrates. Such versatility permits tailored optical designs, including precise control over incident angle, polarization, and light transmission pathways. Early demonstrations using transferred freestanding BFO films have already shown markedly enhanced optoelectronic responses under bending [95]. Future efforts should focus on developing universal, damage‐free film‐release and transfer techniques to enable high‐performance, customizable flexo‐photovoltaic devices.

c. Advanced Strain‐Gradient Engineering

Moving beyond simple macroscopic bending, future research is expected to explore micro‐ and nano‐architectures, such as nanowire arrays, wrinkled patterns, and kirigami/origami‐inspired structures, to program strain gradients at the microscopic scale. Coupling these with stimuli‐responsive materials [96] (e.g., phase‐change VO_2_ or shape‐memory polymers) could lead to smart optoelectronic systems that dynamically tune strain gradients in response to environmental cues like temperature, light, or humidity. Such adaptability opens new avenues for adaptive sensing, optoelectronics, and energy‐harvesting technologies.

d. Multifunctional Coupling and Device Integration

Synergistically combining the flexo‐photovoltaic effect with other complementary mechanisms, such as ferroelectricity, piezoelectricity, and pyroelectricity, could offer the potential for “1 + 1 > 2” multifunctional enhancements [90, 138]. Future device designs may focus on integrated light‐mechanical‐electrical‐thermal energy harvesting and signal‐processing units. Examples include flexible multimodal sensor arrays for biomimetic perception and on‐chip strain‐photovoltaic systems compatible with back‐end CMOS processing.

Through sustained interdisciplinary collaboration spanning materials science, mechanics, photonics, and microfabrication, the flexo‐photovoltaic effect is well positioned to transition from a laboratory‐scale phenomenon into a transformative optoelectronic technology within the coming decade.

## 6 Summary

The flexo‐photovoltaic effect, an emerging optoelectronic phenomenon in which strain gradients induce or enhance photovoltaic or photoconductive responses, has rapidly progressed from a fundamental scientific curiosity into a versatile platform for next‐generation optoelectronic and energy‐harvesting devices. This review has surveyed its manifestations across a broad spectrum of material systems, including conventional oxide perovskites (e.g., SrTiO_3_ and BiFeO_3_), ferroelectric ceramics, various halide perovskites (e.g., MAPbI_3_ and MAPbBr_3_), and low‐dimensional semiconductors such as MoS_2_, CIPS, and InSe. In these materials, mechanically imposed strain gradients break inversion symmetry and generate flexoelectric polarization, which in turn modifies band alignment, facilitates efficient charge carrier separation, and yields photovoltages that can exceed the conventional bandgap limit.

Key experimental advances include the demonstration of giant flexoelectric coefficients under light illumination, strain‐gradient tuning of photocurrent and photovoltage, and the integration of flexo‐photovoltaic response in flexible, freestanding, and composite heterostructures. Significant progress has also been made in coupling this flexo‐photovoltaic effect with other mechanisms, such as ferroelectricity, piezoelectricity, and ion migration, thereby enabling multimodal energy‐harvesting strategies and emerging neuromorphic device functionalities.

Despite these promising developments, some critical challenges still remain. These include the accurate quantification of intrinsic flexoelectric coefficients, material stability under cyclic strain, scalable strain‐gradient engineering, and seamless integration with existing optoelectronic platforms. Looking ahead, the development of freestanding thin films, advanced strain‐gradient‐engineering, theory‐guided material discovery, and multifunctional device integration represents pivotal pathways to overcoming these existing challenges and accelerating the transition toward practical applications. Ultimately, the flexo‐photovoltaic effect not only enriches our understanding of strain‐photonic‐charge coupling but also opens new avenues for high‐efficiency, adaptive, and mechanically responsive energy and optoelectronic technologies beyond the constraints of conventional photovoltaic and photodetection paradigms.

## Conflicts of Interest

The authors declare no conflicts of interest.

## Data Availability

Data sharing not applicable to this article as no datasets were generated or analyzed during the current study.
